# The Effect of Surveillance and Appreciative Inquiry on Puerperal Infections: A Longitudinal Cohort Study in India

**DOI:** 10.1371/journal.pone.0087378

**Published:** 2014-01-30

**Authors:** Julia Hussein, K. V. Ramani, Lovney Kanguru, Kalpesh Patel, Jacqueline Bell, Purvi Patel, Leighton Walker, Rajesh Mehta, Dileep Mavalankar

**Affiliations:** 1 Immpact, University of Aberdeen, Aberdeen, Scotland; 2 Indian Institute of Management, Ahmedabad, India; 3 Indian Institute of Public Health, Gandhinagar, India; University of Western Australia, Australia

## Abstract

**Objective:**

To evaluate the effects of an intervention comprising surveillance and an organisational change called Appreciative Inquiry on puerperal infections in hospitals in Gujarat state, India.

**Methods:**

This longitudinal cohort study with a control group was conducted over 16 months between 2010 and 2012. Women who delivered in six hospitals were followed-up. After a five month pre-intervention period, the intervention was introduced in three hospitals. Monthly incidence of puerperal infection was recorded throughout the study in all six hospitals. A chi-square test and logistic regression were used to examine for associations, trends and interactions between the intervention and control groups.

**Findings:**

Of the 8,124 women followed up, puerperal infections were reported in 319 women (3.9%) over the course of the study. Puerperal sepsis/genital tract infections and urinary tract infections were the two most common puerperal infections. At the end of the study, infection incidence in the control group halved from 7.4% to 3.5%. Levels in the intervention group reduced proportionately even more, from 4.3% to 1.7%. A chi-square test for trend confirmed the reduction of infection in the intervention and control groups (p<0.0001) but the trends were not statistically different from one another. There was an overall reduction of infection by month (OR = 0.94 95% CI 0.91–0.97). Risk factors like delivery type, complications or delivery attendant showed no association with infection.

**Conclusion:**

Interruption of resource flows in the health system occurred during the intervention phase, which may have affected the findings. The incidence of infection fell in both control and intervention groups during the course of the study. It is not clear if appreciative inquiry contributed to the reductions observed. A number of practical and methodological limitations were faced.

**Trial Registration:**

Controlled-Trials.com ISRCTN03513186

## Introduction

Maternal mortality and morbidity from puerperal sepsis and other infections related to childbirth directly reflect aspects of the quality and safety of obstetric services. Puerperal sepsis is one of the leading causes of maternal mortality worldwide and the second most common cause of maternal death after postpartum haemorrhage in Asia and Africa, accounting for as much as 15% of deaths [1]. Other obstetric puerperal infections, such as genital tract infections, wound infections and urinary tract infections following delivery may be less devastating but are nevertheless responsible for ill health and slow recovery of the mother in the postpartum period. Puerperal infections are directly associated with early onset neonatal sepsis and can also affect newborn wellbeing indirectly, causing difficulties for example in breastfeeding and by interfering with mother and child bonding.

Puerperal infections are usually introduced during labour and childbirth. Apart from the risks of unhygienic practices by birth attendants in the community, infections may also be a result of poor quality of care received in health facilities. In some developing countries, the uptake of delivery care in health facilities is increasing, with consequent risks of health facility acquired infections. Typical infection control problems in health facilities include low awareness of the problem amongst health personnel, poor antibiotic prescribing, lack of basic water and sanitation infrastructure, absence of surveillance data and inadequate laboratory services [2]–[4].

Current recommendations for infection control involve the implementation of multiple approaches in health facilities, including the use of guidelines, protocols, education, training, feedback, surveillance and organisational change [3], [4]. Such multimodal strategies have also been highlighted by the World Health Organization’s Global Patient Safety Challenge [5]. A few studies have demonstrated reductions in infection rates using multimodal approaches [6], [7], but there have been no evaluations of interventions to reduce puerperal infections in resource constrained settings [8].

India’s maternal mortality ratio has been falling since 1990 [9], [10] with current levels believed to be as low as 200 per 100,000 live births. At national level, a sixth of maternal deaths are reported to be due to puerperal sepsis [11], [12] although sub-national studies have estimated that puerperal sepsis may cause as much as 42% of maternal deaths [13], [14]. In 2009, we conducted a needs assessment in 20 maternity units in Gujarat state which identified the need for guidelines and standards for infection control, improved function of infection control committees, documentation, feedback and audit [15]. The findings of the needs assessment, global recommendations for multimodal interventions and the paucity of studies on infection control in maternity units formed the basis of our study and informed our intervention. We used a motivational organisational change intervention known as Appreciative Inquiry (AI) and aimed to assess its effects on puerperal infections in hospitals. The concept of AI was originally developed as a management technique in the 1980s [16] to promote organisational creativity and learning [17]. Its focus is on what an organization does well and builds upon this, rather than on negative aspects. In maternal health programmes, it has been used to improve the quality of obstetric care in countries such as Bangladesh, India and Nepal [18], [19] as well as in industrialised countries as a quality improvement intervention [20]. Reports of its effects have been in improved motivation, better teamwork and respectful interactions [18], [20] but improvements in service delivery or health outcomes have yet to be demonstrated. We set out to test the effect of AI on infection rates as it was expected to lead to locally formulated, creative, multimodal strategies for action and a sense of team ownership. Our needs assessments [15] suggested that infection control committees for example, sometimes do not function well because they involve common meetings between staff at different levels of hierarchy. We postulated that AI might work by breaking down hierarchal barriers and improving team working which would lead to changes in behaviour and practice (for instance by hand washing, improved cleaning procedures, reducing unnecessary interventions like caesarean section or prescribing antibiotics based on evidence).

## Methods

The protocol for this study and supporting ORION checklist are available as supporting information; see Checklist S1 and Protocol S1. The study was registered on the Current Controlled Trials register ISRCTN03513186.

### Ethics Statement

Ethical approval was obtained from the Research and Publications Committee of the Indian Institute of Management Ahmedabad and permissions for the study from the Gujarat Government Department of Health. Written consent was obtained from all study participants in the local language. If the participant was illiterate, the study was explained, the consent form read out and the woman asked to make a thumbprint on the study form in the presence of a witness.

### Study Setting and Site Selection

This study was conducted in Gujarat state, with a population of about 50 million. The maternal mortality ratio in this state is 148 per 100,000 live births [21]. Uptake of maternity services is increasing and higher than the national average, with more than 53% of women delivering in health facilities and 61% of deliveries receiving a postnatal check [9]. The improving use of maternity facilities in Gujarat highlights the importance of nosocomial infection control. The formal health care system comprises the primary level (primary health centres and sub-centres), secondary level (first referral units and community health centres), tertiary level (district hospitals), multi-specialty state hospitals and medical college hospitals. Hospitals of interest in our study were at the secondary and tertiary levels. The state has approximately 500 secondary and tertiary care units [22]. Delivery care in these hospitals is provided by specialist obstetricians, general physicians and nurses.

Our criteria for selection of hospitals were (a) those with a high number of deliveries (over 1,000 deliveries per year or as close as possible) (b) routinely able to deal with at least some obstetric complications (c) representation of government and private non profit hospitals (d) willingness to be involved in the study, and (e) covering a population that would make home visits feasible. Six facilities closely matching the criteria were listed. Four were government hospitals and two were private non profit hospitals. The two busiest facilities were paired and a ‘toss of a coin’ determined which was allocated to control and intervention group, with one government hospital and one private hospital in each group. The second pair of hospitals with the next highest number of deliveries was allocated so that the control and intervention groups each contained one private hospital. The final pair of hospitals was then assigned, finally allowing a private facility in each group and roughly similar overall sample sizes. The intervention group comprised facilities H1–H3 and the control group H4–H6 (Table 1).

**Table 1:** Characteristics of study hospitals.

Facility type	Intervention group			Control group		
	H1	H2	H3	H4	H5	H6
	Governmentsub-district level	Government sub-district level	Private	Governmentsub-district level	Governmentdistrictlevel & academic	Trust
**Number of deliveries** **per annum**	1257	900	3200	650	4920	900
**Approximate staff ratio**						
Total number of doctors inthe hospital: maternity	7∶3	9∶5	9∶2	7∶3	23∶2+ including 2 residents	11∶3
Total number of nurses inthe hospital: maternity	7∶7	6∶6	14∶14	6∶6	163∶13	8∶8

### Study Design, Population and Data Collection

The study was a prospective, controlled, longitudinal cohort study with a predefined protocol. Women who delivered in the three hospitals H1–H3 were ‘exposed’ to the intervention described below. The ‘control’ group of women delivered in hospitals H4–H6 where the intervention did not take place. Women in both intervention and control groups who received delivery care in health facilities were identified at the time of admission and followed for 42 days post partum, to determine if they developed a puerperal infection.

A pilot phase to train data collectors and test the instruments was carried out in September and October 2010. The training focused on questionnaire orientation and interview techniques in various settings. Some simplifications to the questionnaire were made. Follow up home visits were found to be feasible, provided study participants lived within 20 km of the study hospital.

The main study was conducted over 16 months from 1^st^ November 2010 to 29^th^ February 2012. The study population comprised women who delivered in the intervention or control hospitals during this period. Women over 28 weeks gestation who delivered a live or stillborn baby or who delivered a baby (in any location be it in the community, a study site or a non-study hospital) and were subsequently admitted to the study hospitals with the placenta undelivered, were eligible for inclusion. Women admitted to the study hospitals after delivery of the placenta were not included. Pregnant women who delivered before 28 weeks gestation or with a miscarriage or abortion were also excluded.

**Figure 1:**
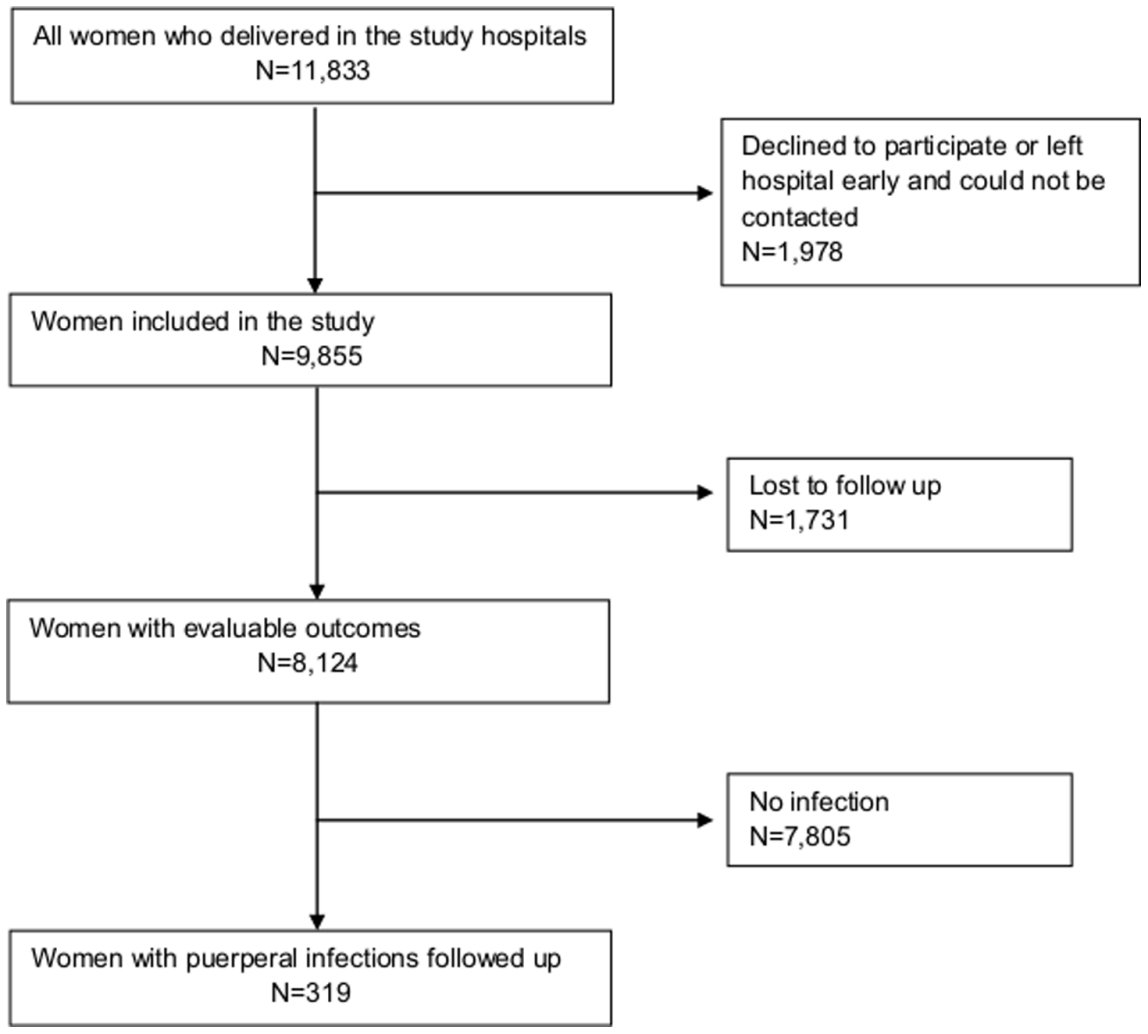
CONSORT diagram illustrating follow up of study population.

A data collector was assigned to each study hospital to visit labour and post natal wards every morning. The data collectors were nursing assistants with basic knowledge of nursing skills and experience in hospitals. Using the hospital registers, the data collector established the number of women who had delivered the previous day. The number of women who declined to participate, who left the hospital early with no contact details before the data collector could meet with them, or who were lost to subsequent follow up (unable to trace the address or living too far away), were recorded (Figure 1). For women who had left the hospital early, if an address from the hospital register was available, attempts were made to interview these women at home. For all women included in the study, a unique case identification number was assigned.

Puerperal sepsis and other puerperal infections as specified in ICD-10 codes 085 and 086 [23] including infections of obstetric surgical wounds, genital tract and urinary tract infections following delivery were the outcomes of interest. The identification of infection was based on signs and symptoms. Based on the ICD-10 codes and a review of standard obstetric textbooks [24]–[27], a questionnaire was developed to elicit signs or symptoms of pain, fever, abnormal vaginal discharge, delay in uterine involution, frequency or urgency of micturation, wound redness, swelling, discharge and location of infection. The signs and symptoms in the questionnaire are represented in Table 2. Antibiotic use was recorded. In the hospital, the questionnaire was administered daily by the data collector to obtain information from the study participants, nurses and doctors. Women were asked to provide an address and telephone contact to allow a data collector to interview them at home. After discharge from the hospital, women were followed up in person at designated time points (as close to the 7^th^, 28^th^ and 42^nd^ post-partum day as possible) and through phone calls as needed. Additional home visits were made if a participant described symptoms of, or reported being treated, for puerperal infection.

**Table 2:** Definitions used to identify puerperal infections in study.

Condition	Signs and symptoms
1. **Puerperal sepsis**: Infection of the genital tractoccurring at any time between onset of rupture ofmembranes or labour; and the 42^nd^ day postpartumin which two or more of the following are present [24]:	a. Pelvic pain
	b. Fever i.e. oral temperature 38.5°C/101.3°F or higher on any occasion
	c. Abnormal vaginal discharge, e.g. presence of pus
	d. Abnormal smell/foul odour of discharge and
	e. Delay in the rate of reduction of size of the uterus (<2 cm a day for the first 8 days)
**2. Genital tract infection:** Post partum purulentor malodourous lochia and at least one of a to c [26]:	a. Pelvic pain
	b. Abdominal pain
	c. Delay in the reduction of the size of the uterus (<2 cm a day for the first 8 days)
**3. Urinary tract infection:** Symptoms developingpost partum with pain on micturition, and atleast one of a and b [25]:	a. Cloudy or discoloured urine
	b. At least one of the following (i) Increased frequency (ii) Urgency (iii) Hesitancy (iv) Dribbling (v) Purulent urethral discharge
**4. Episiotomy or perineal tear infection:**Acquired during the patient’s most recentdelivery with at least two of symptoms a to cor one of a to c plus at least 2 of symptomsd to g [25], [27]:	a. Discharge from the wound
	b. Purulent discharge
	c. Wound begun to open up
	d. Bruising around the wound
	e. Redness around the wound
	f. Swelling around the wound
	g. Tenderness around the wound
**5. Caesarean section wound infection:**Delivery of most recent baby by caesareansection and a+b; or one of a or b and two ofc to f; or at least three of c to f [27]:	a. Wound begun to open
	b. Purulent discharge
	c. Bruising around wound
	d. Redness around wound
	e. Swelling around the wound
	f. Tenderness around the wound

We divided the study into three phases: 5 months pre-intervention (November 2010–March 2011); 8 months intervention (April 2011–November 2011) and 3 months post-intervention (December 2011–February 2012). Our protocol had planned for a minimum of seven months intervention to cross over the seasonal change to the wet season (June-September) which might affect infection rates. During the pre-intervention phase, data was collected from women in hospitals and at home in all the six study sites. In February 2011, the AI specialist and the project researchers conducted a visit to observe how the study hospitals functioned, particularly the labour and delivery areas. A few hospital staff and women in the labour wards were informally interviewed. Although all hospitals had staff members who were designated as responsible for infection control, formal committees were not routinely functioning. The purpose of this visit was to facilitate the design of the AI process. Data on infection rates were not fed back to any of the six hospitals until after the study was completed.

### Intervention

**Figure 2:**
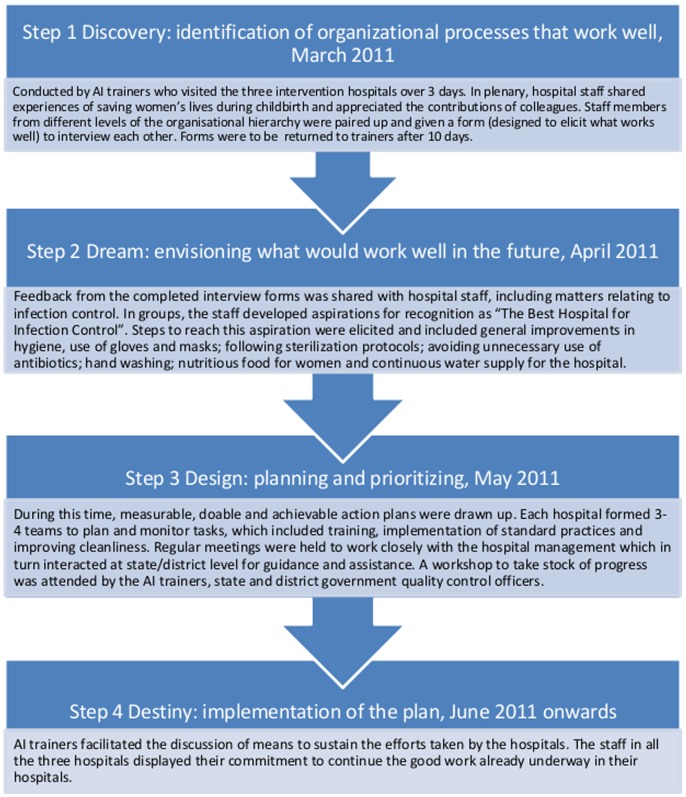
Appreciative inquiry, description of the study intervention and key events.

The intervention comprised a series of workshops and activities conducted by hospital staff for planning, prioritisation and implementation using AI. This change and development focused on positive aspects, i.e. what is done well and what works, rather than trying to fix what doesn’t. The intervention comprised four main steps detailed in Figure 2. At the end of March 2011, the AI intervention was initiated with a workshop attended by staff of H1, H2 and H3. State/district government officers from the quality control divisions in the health departments also attended and presented existing infection control policies and guidelines. The workshop also provided an opportunity for the participants to develop an overall understanding of AI and how the process differs from traditional approaches to problem solving. Detailed AI sessions at these hospitals followed and action plans for infection control were developed by May 2011 for implementation from June.

In the control sites, hospital staff members were aware that data was being collected on puerperal infections, but they received no feedback on the project during the course of the study. The control sites continued to receive routine visits from government officers responsible for quality control. The research team also visited the control hospitals to ensure data continued to be collected.

An unexpected event occurred in June. State government hospitals (including those in our study in both control and intervention groups) faced severe financial constraints due to governance issues in the allocation of national health funds. This resulted in shortages of supplies like gloves and other consumables, which affected government hospitals in both intervention and control groups. Staff disruptions also occurred at the same time. The medical superintendent in one of the intervention hospitals went on three months leave, while four permanent nurses in another intervention hospital were transferred. Temporary staff appointed in these hospitals did not take the same amount of interest taken by the regular staff in implementing the action plans. In the next four months, visits to the 2 intervention government hospitals to monitor the implementation of infection control measures were undertaken by the research team to motivate the hospital staff to implement the action plan with some success despite the constraints. The interventions in the third intervention hospital were going on smoothly, requiring no additional visits. The disruptions eased off by September 2011, resulting in unencumbered implementation of action plans from October onwards. The intervention period ended in November 2011 followed by a three month post intervention period. Given the interruption in implementation of action plans during the intervention period, prolongation of the intervention phase was considered but could not be accommodated within the study funds available.

### Analysis

The analysis was conducted as intention to treat at both hospital and participant level. Taking an expected infection rate of 10%, we estimated that inclusion of the 6 selected hospitals would allow us to detect a reduction in infection of 20% with 80% power at 5% significance level assuming random effects and accounting for clustering. Women with more than one infection recorded were only counted once in the analysis. We compared the distribution of socio-economic, pregnancy and delivery characteristics and the proportion of women infected over time between control and intervention groups. Logistic regression was used to test the association between infection and each delivery characteristic independently and with combinations of variables. Trends in levels of infection in intervention and control hospitals were identified by period using a chi-square test of trend, and by month of delivery using a logistic regression model. Two-sample tests of proportions were used to check imbalances in characteristics of women between control and intervention groups. STATA (version 12) was used to conduct the analysis.

Reporting of the study adhered to ORION guidelines for reporting of infection control studies [28] and used the CONSORT flowchart [29]. Minor deviations from the study protocol were: a delay in commencing the study by six months to ensure adequate preparation for the study, the inclusion of six, rather than the original seven hospitals as sufficient deliveries were expected from six hospitals, and follow up of women by telephone interview only (the original plan was follow up by using a self reporting symptom card and telephone interviews).

## Findings

Of the 11,833 women who delivered in the study sites during the study period, 8,124 women were followed up to the 42^nd^ day post-partum (Figure 1). Table 3 summarises the characteristics of women in control and intervention groups. Most women were aged between 20 and 30 years and were of parity three or less. Differences in poverty (through possession of the BPL, the ‘below poverty line’ card issued by the government) were observed between intervention and control groups. A greater proportion of women received episiotomies in the intervention group but caesarean sections were more frequent in the control group. Women in the intervention group had shorter hospital stays.

**Table 3:** Socio-economic, pregnancy and delivery characteristics of women in the sample.

	Intervention N = 4868	Control N = 3256	p-value for difference
**Age**			
Mean years (SD)	24.8 (3.85)	24.6 (3.93)	0.018
**Years of Education**			
Mean years (SD)	4.7 (4.34)	4.4 (4.41)	0.007
**‘Below Poverty Line’ card**			
n (%)	2352 (48.32)	1380 (42.38)	<0.001
**Parity**			
Mean births (SD)	2.1 (1.12)	2.0 (1.10)	0.031
**Mode of delivery** n (%)			
Normal	2350 (48.27)	1672 (51.35)	0.007
Normal (with episiotomy)	2006 (41.21)	1114 (34.21)	<0.001
Assisted normal	46 (0.94)	24 (0.74)	0.320
C-Section	466 (9.57)	446 (13.70)	<0.001
**Delivery complications**			
n (%)	563 (11.57)	317 (9.74)	0.009
**Delivery of: baby-placenta** n (%)			
Doctor-Doctor	718 (14.75)	472 (14.50)	0.750
Nurse-Nurse	3571 (73.36)	2471 (75.89)	0.010
Both	569 (11.69)	298 (9.15)	<0.001
Other	10 (0.21)	15 (0.46)	0.042
**Delivery outcome** n (%)			
Still Birth	88 (1.81)	67 (2.06)	0.652
**Antibiotic given during or after delivery**			
n (%)	3706 (76.13)	2496 (76.66)	0.582
**Hospital stay days** n (%)			
0–3 day(s)	4655 (95.62)	2642 (81.14)	<0.001
4–7 days	87 (1.79)	399 (12.25)	<0.001
≥8 days	4 (0.08)	62 (1.90)	<0.001

Table 4 shows the percentage of women infected in the control and intervention groups. A total of 319 women contracted puerperal infections during the study period. The overall incidence of infection in the pre-intervention period was 5.7%, although the range varied considerably between hospitals, from just over 1% to 17%. Infection incidence gradually reduced in both intervention and control groups over the three phases of the study. In the control hospitals, incidence halved from 7.4% to 3.5% and reduced even more from 4.3% to 1.7% in the intervention hospitals over the whole 16 month study period. However, the percentage point decrease in the control group (3.9 percentage points) was greater than in the intervention group (2.6 percentage points). The chi-square test for trend showed a statistically significant (p<0.0001) downward trend of infection incidence overall. Logistic regression confirmed the lower infection incidence in the intervention group compared with the control group (OR 0.60 95% CI 0.39–0.92) and an overall reduction in infection by month (OR 0.94 95% CI 0.91–0.97) but no difference in the trend between intervention and control groups (p = 0.37).

**Table 4:** Puerperal infections in women.

N = 8124	Intervention n (%)				Control n (%)				All hospitals
	H1	H2	H3	H1,H2,H3 total	H4	H5	H6	H4,H5,H6 total	
**Pre-intervention** *(Nov. 2010–Mar. 2011)*	14 (3.43)	31 (16.76)	8 (1.24)	**53 (4.28)**	30 (14.71)	35 (6.28)	12 (4.21)	**77 (7.36)**	**130 (5.69)**
**Intervention** *(Apr. 2011–Nov. 2011)*	39 (6.25)	19 (6.48)	8 (0.47)	**66 (2.51)**	22 (7.05)	52 (5.56)	12 (3.13)	**86 (5.28)**	**152 (3.57)**
**Post intervention** *(Dec. 2011–Feb. 2012)*	9 (3.78)	4 (3.42)	4 (0.62)	**17 (1.69)**	6 (4.88)	11 (3.65)	3 (1.92)	**20 (3.45)**	**37 (2.34)**
**Total**	**62 (4.88)**	**54 (9.08)**	**20 (0.67)**	**136 (2.79)**	**58 (9.08)**	**98 (5.47)**	**27 (3.28)**	**183 (5.62)**	**319 (3.93)**

**Figure 3:**
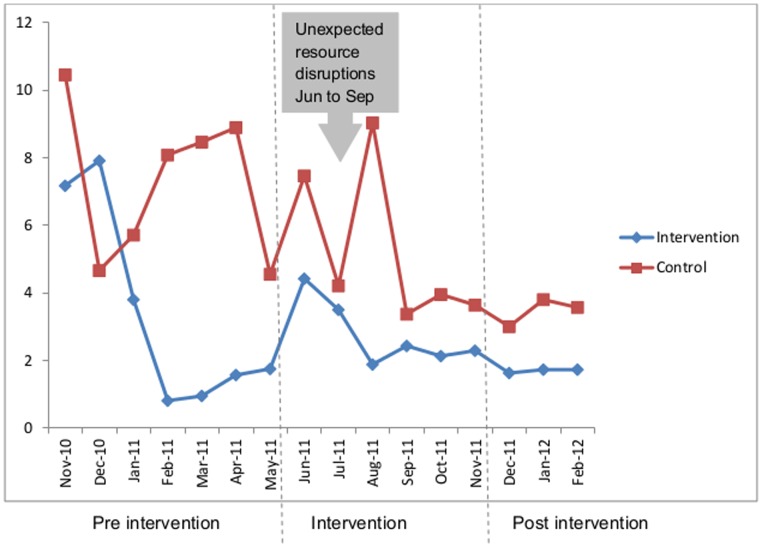
Percentage of women infected by month.

Figure 3 illustrates the fall in percentage of women infected during the study period by month in control and intervention groups. In the intervention group there was a rapid reduction in infection before the intervention phase started. When financial constraints and staff transfers were experienced in the government hospitals (June to August 2011), a sudden increase in incidence of infection ocurred. Patterns of change were more erratic in the control group. Examination of the trends in individual hospitals (data not shown) between June and August 2011 show that infection increased in some government hospitals (H1, H2 and H4) but were not marked in any of the other government or private hospitals.

Four types of puerperal infections were recorded – puerperal sepsis/genital tract infections, urinary tract infection, perineal and caesarean section wounds. Of the infections across all hospitals in the pre-intervention period, 3.6% were puerperal sepsis, nearly 2% urinary tract infections and the remainder were wound infections. Puerperal sepsis was consistently the most common infection recorded in intervention and control groups and in all periods of the study, except in the intervention group during the intervention period when it was equal to urinary tract infections at 1.2% each.

Associations between infection and hypothesised risk factors of delivery type, complications, attendant at delivery, outcome of delivery (live birth or stillbirth), antibiotic use and hospital stay days were investigated. Logistic regression testing the relationship independently and cumulatively showed no significant correlations for any of the risk factors (Table 5).

**Table 5:** Logistic regression looking at the relationship between infection and selected risk factors measured cumulatively.

	Odds ratio	p>[z]	95% Confidence Interval
**Mode of delivery**	1.03	0.72	0.87–1.23
**Complications**	1.05	0.23	0.97–1.15
**Person conducting delivery**	0.97	0.78	0.78–1.20
**Delivery outcome**	1.19	0.67	0.55–2.58
**Antibiotic**	1.23	0.16	0.92–1.66
**Hospital stay days**	1.17	0.13	0.96–1.44

## Discussion

There are few sources of data on infection in India available to compare our study against. In 2009, we recorded rates of 3–5% in maternity units [15] while others have reported infection rates of 6% [30] and 18% [31] in non-intensive care surgical settings. These figures suggest that the levels of infection we recorded are plausible. Our study is one of the first instances of infection surveillance in maternity units in India.

The highest levels of infection were found in government hospitals with fewer than 900 deliveries per year and the lowest levels in government and in private hospitals with the same or a higher volume of work. No clear patterns were discernible with type of hospital, workload or staffing levels. Low staffing levels were noted. As few as 2 doctors and 13 nurses were available for the busiest maternity unit (5,000 deliveries every year). This government hospital (H5) did not have especially high infection rates, yet another government hospital (H2) with better staff to delivery ratios had the highest infection rate of all the six hospitals. The high use of antibiotics may have affected patterns of infection across hospitals and lowered levels of infection. Antibiotics were given to at least 60% of women in all except one hospital (H1), which despite its relatively low use of antibiotics (22%) did not have particularly high infection rates. High antibiotic use was also documented in our previous needs assessment study [15] where we found that over-prescribing and routine administration of antibiotics in normal deliveries was common.

The control hospitals had higher infection incidence than the intervention hospitals during the pre-intervention and intervention phases. The disproportionate effect of the busier hospitals may be one explanation. This group also comprised women who were less poor and had more caesarean sections.

Puerperal infections halved in the control group and proportionately reduced even more in the intervention group during the course of the study. Our starting hypothesis was that AI could reduce infection by improving for example, team working, the functionality of infection control committees, changes in behaviours or practice (e.g. hand washing) and reducing unnecessary interventions (e.g. overuse of caesarean section or antibiotics). In our study, we tracked antibiotic use and caesarean section rate (data not shown) by hospital and by month, but did not demonstrate any trends related to the introduction of AI. These are however only two and also somewhat unrefined indicators of practice change, but it was not feasible to measure the many other aspects. A statistically significant difference in trend between intervention and control groups was not found, so it is not possible to determine conclusively if AI resulted in the larger decline observed in the intervention group. We raise the possibility that a decrease in infection levels may be easier to achieve from a higher point (i.e. from 7% to 3% in the control) than it is to decrease levels from a lower point (i.e. to 2% or less in the intervention group).There are other important explanations for the observed reductions. The Hawthorne effect is well recognised in infection surveillance studies and is evidenced by the drop in infection rates in the pre-intervention phase. The hospital staff in both control and intervention sites may have changed their behaviour as a result of knowing they were being studied, rather than because of the intervention. Surveillance of infection took place in both groups but only the intervention group received AI interventions. Control and intervention hospitals received visits from researchers for data collection. We minimised the Hawthorne effect by having several months pre-intervention to allow stabilisation and by not feeding back findings until the study was complete. Contamination across control and intervention groups could not be eliminated as the district health officers involved in the AI activities were equally responsible for control and intervention hospitals and the discussions may have raised their awareness of infection control generally which were then reflected in the control sites. The observational nature of a longitudinal cohort study also makes it subject to a number of biases. To reduce the risk of confounding effects, we assigned hospital sites to control and intervention groups randomly but the small numbers (only three hospitals in each group) means that limitations of clustering remained. Although we paired hospitals using some characteristics, matching of individual women’s characteristics or blinding could not be incorporated within the scope of the study and some imbalances in women’ characteristics were noted (Table 3), so selection biases could have existed.

Various other limitations should be noted. The definitions of the various puerperal infections are based on a range of signs and symptoms which are not necessarily reliably assessed. Microbiological tests were not used to confirm the signs and symptoms of infection. Over and under-reporting are both possibilities. Minor infections, spontaneous resolution of symptoms and loss to follow up may have led to cases being missed. Our intention was to capture nosocomial (hospital acquired) infection rates but it was not possible to determine where the infections we recorded were contracted. The study was not powered to detect changes at the relatively low levels of infection recorded. There are no other studies in India of infection in maternity care to ascertain whether the downward trend we observed in our study is comparable to other experiences.

Despite these limitations, the observed reduction in infection observed underscores the potential value of monitoring infection in maternity units. Our data captured changes in infection rates when the government financial restrictions and staffing changes occurred, suggesting that the occurrence of infection could be sensitive to these systems variations. The gap between infection rates in the control and intervention groups had narrowed by the post-intervention phase.

## Conclusion

The lack of data and knowledge on puerperal infections is part of the knowledge gap which this study aspired to fill. Despite reported reductions in the proportion of deaths from puerperal sepsis worldwide, infection in maternity units continues to burden health services and cause ill health among women and babies. Our study suggests that infection surveillance may reduce puerperal infections in women who deliver in maternity units. The added effect of introducing a motivational organisational change process called AI is possible, but not conclusive. In light of the methodological constraints and uncertainty of findings from the study, we recommend a renewal of interest in infection control research. The establishment of large collaborative groups at national and international level may help in bringing resources to what is otherwise a neglected area. Such groups may bring together diverse disciplinary perspectives that can contribute to methodological advances in the study of the complex organisational and behavioural interventions not amenable to conventional quantitative research approaches. In addition to research considerations, monitoring of infection rates should become a priority in all maternity units and may be a preventive intervention in itself. Simple, robust means of accurately diagnosing different types of puerperal infections are needed alongside development of microbiological diagnostic capacity in low and middle income countries. Antibiotic overuse, poor staffing levels and government procedural delays are some of the factors captured in this study which need to be considered in order to improve the quality and safety of health facility care in India.

## Supporting Information

Checklist S1 Orion statement table.
(DOCX)

Protocol S1 Original study protocol.
(DOC)
