# Supplementary material for: The Effect of Surveillance and Appreciative Inquiry on Puerperal Infections: A Longitudinal Cohort Study in India
Source: PLoS One. 2014 Jan 30;9(1):e87378. doi: 10.1371/journal.pone.0087378 (PMC3907541; doi:10.1371/journal.pone.0087378)
Supplement: Checklist S1 — Orion statement table. (DOCX) [file pone.0087378.s001.docx]

# **ORION Checklist of items to include when reporting an outbreak or intervention study of a nosocomial organism**

Supplement to manuscript: The effect of surveillance and appreciative inquiry on puerperal infections: A longitudinal cohort study in India (Hussein et al)

|  | **Item Number** | **Descriptor** | **Section reference in manuscript** |
| --- | --- | --- | --- |
| **Title & Abstract** | 1 | Description of paper as outbreak report or intervention study.  Design of intervention study (eg Randomised Controlled Trial , Cluster Randomised Controlled Trial, Interrupted Time Series, Cohort study etc).  Brief description of intervention and main outcomes. | Introduction section |
| **Introduction**  Background | 2 | Scientific and/or local clinical background and rationale.  Description of organism as epidemic, endemic or epidemic becoming endemic. | Study design, population and data collection section.  Description of organism n/a |
| Type of paper | 3 | Description of paper as Intervention study or an Outbreak Report.  If an outbreak report, report the number of outbreaks. | An intervention study. Study design, population and data collection section |
| Dates | 4 | Start and finish dates of the study or report. | Study design, population and data collection section |
| Objectives | 5 | Objectives for outbreak reports. Hypotheses for intervention studies | End of introduction section |
| **Methods**  Design | 6 | Study design. Use of EPOC classification recommended (RCT or CRCT, CBA, or ITS)  Whether study was retrospective, prospective or ambidirectional.  Whether decision to report or intervene was prompted by any outcome data.  Whether study was formally implemented with predefined protocol and endpoints. | Study design, population and data collection section |
| Participants | 7 | Number of patients admitted in study or outbreak. Summaries of distributions of age and lengths of stays. If possible, proportion admitted from other wards, hospitals, nursing homes or from abroad. Where relevant, potential risk factors for acquiring the organism. Eligibility criteria for study. Case definitions for outbreak report. | Study design, population and data collection section, Figure 1 and Table 3 |
| Setting | 8 | Description of the unit, ward or hospital and, if a hospital, the units included.  Number of beds, the presence and staffing levels of an infection control team. | Table 1 |
| Interventions | 9 | Definition of phases by major change in specific infection control practice (with start and stop dates). A summary table is strongly recommended with precise details of interventions, how and when administered in each phase. | Intervention section, Figure 2 and 3 |
| Culturing & Typing | 10 | Details of culture media, use of selective antibiotics and local and /or reference typing. Where relevant, details of environmental sampling. | n/a |
| Infection-related outcomes | 11 | Clearly defined primary and secondary outcomes (eg incidence of infection, colonisation , bacteraemia) at regular time intervals (eg daily, weekly, monthly) rather than as totals for each phase, with at least three data points per phase and, for many two phase studies, 12 or more monthly data points per phase. Denominators (eg numbers admissions or discharges, patient bed days). If possible, prevalence of organism and incidence of colonisation on admission at same time intervals. Criteria for infection, colonisation on admission and directly attributable mortality.  For short studies or outbreak reports, use of charts with duration patient stay & dates organism detected may be useful (see text) | Study design, population and data collection section, Table 2, Figure 3 |
| Economic outcomes | 12 | If a formal economic study done, definition of outcomes to be reported, description of resources used in interventions, with costs broken down to basic units, stating important assumptions. | n/a |
| Potential Threats to internal validity | 13 | Which potential confounders were considered, recorded or adjusted for (eg: changes in length of stay, case mix, bed occupancy, staffing levels, hand-hygiene compliance, antibiotic use, strain type, processing of isolates, seasonality).  Description of measures to avoid bias including blinding & standardisation of outcome assessment & provision of care. | Table 3, analysis section and discussion section |
| Sample size | 14 | Details of power calculations, where appropriate | Analysis section |
| Statistical methods | 15 | Description of statistical methods to compare groups or phases. Methods for any subgroup or adjusted analyses, distinguishing between planned and unplanned (exploratory) analysis. Unless outcomes are independent, statistical approaches able to account for dependencies in the outcome data should be used, adjusting, where necessary, for potential confounders.  For outbreak reports statistical analysis may be inappropriate. | Analysis section |
| **Results**  Recruitment | 16 | For relevant designs the dates defining periods of recruitment and follow-up. A flow diagram is recommended to describe participant flow in each stage of study. | Study design and intervention sections and Figure 1 |
| Outcomes & estimation | 17 | For the main outcomes, the estimated effect size and its precision (usually using confidence intervals). A graphical summary of the outcome data is often appropriate for dependent data (such as most time series). | Figure 3 and Table 4 |
| Ancillary analyses | 18 | Any subgroup analyses should be reported and it should be stated whether or not it was planned (specified in the protocol) and possible confounders adjusted for | n/a |
| Adverse events | 19 | Pre-specified categories of adverse events and occurrences of these in each intervention group . This might include drug side effects, crude or disease specific mortality in antibiotic policy studies or opportunity costs in isolation studies. | n/a |
| **Discussion**  Interpretation | 20 | For intervention studies an assessment of evidence for/against hypotheses, accounting for potential threats to validity of inference including regression to mean effects and reporting bias.  For outbreak reports, consider clinical significance of observations and hypotheses generated to explain them. | Discussion section |
| Generalisability | 21 | External validity of the findings of the intervention study i.e. to what degree can results be expected to generalise to different target populations or settings. | Discussion section |
| Overall evidence | 22 | General interpretation of results in context of current evidence. | Discussion and conclusion section |

**Abbreviations:** RCT: randomised controlled trial CRCT : Cluster Randomised Controlled Trial CBA: controlled before and after study ITS: interrupted time series
