# Supplementary material for: The Effect of Surveillance and Appreciative Inquiry on Puerperal Infections: A Longitudinal Cohort Study in India
Source: PLoS One. 2014 Jan 30;9(1):e87378. doi: 10.1371/journal.pone.0087378 (PMC3907541; doi:10.1371/journal.pone.0087378)
Supplement: Protocol S1 — Original study protocol. (DOC) [file pone.0087378.s002.doc]

**An infection control intervention study:**

**Using infection control as an entry point for improving the quality of delivery care and strengthening health systems in developing countries**

Operational Protocol

Version 29^th^ July 10


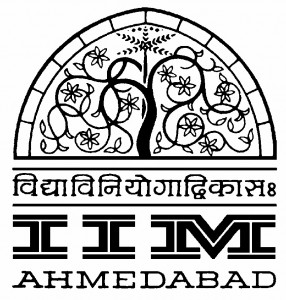


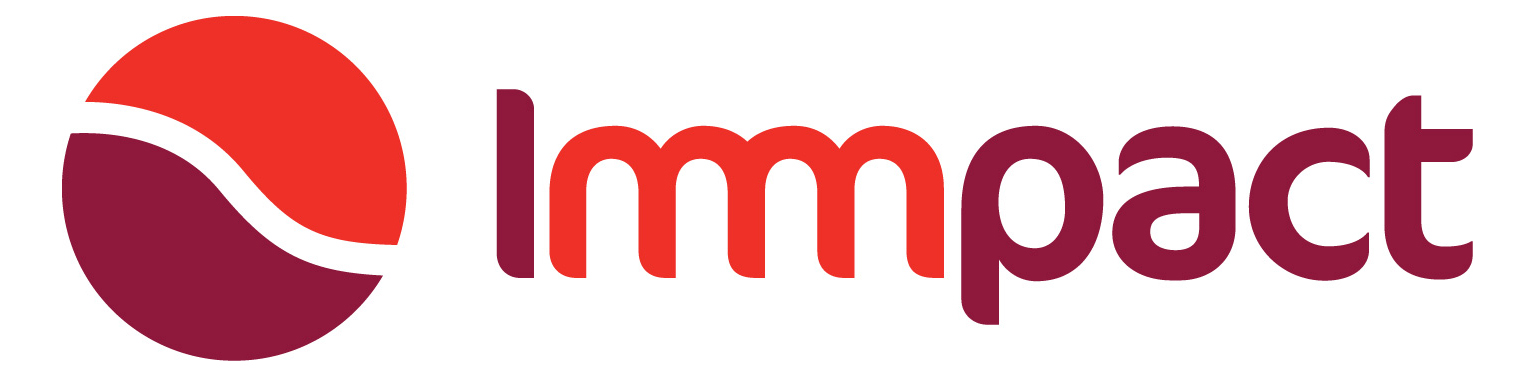


1. **BACKGROUND**
   1. **Puerperal Sepsis and its part in maternal mortality**

The Fifth Millennium Development Goal – to improve maternal health - has been acknowledged as the most seriously off target of the eight goals. Although concern to reduce maternal mortality has existed for a number of years, it has been amplified recently after the publication of 2005 estimates showing minimal decline since 1990 [1]. In the period remaining up to 2015, it is predicted that there will be concerted efforts by implementing agencies to scale-up and speed-up the effective coverage of maternity services alongside broader reproductive health care. Such reproductive health services would need to provide equitable, timely and affordable access to appropriate healthcare. There is thought to be a positive link between the status of maternal health, the effective coverage of maternity services and the functionality of the overall health system of a country. This presumed relationship is reflected in calls both to use maternal health indicators as proxies of the functionality of a health system, and, conversely, to strengthen health systems as the essential route to achieving MDG5.

Direct causes are responsible for the majority of maternal deaths in low income countries. Behind post partum haemorrhage (PPH) and unsafe abortion, infection is the commonest cause of death following childbirth, accounting for as much as 15% of the half million maternal lives lost annually [4]. Many of these deaths are avoidable and the figure would be reduced with improved access to care and improved quality of care.

Given the scale of the problem and the number of deaths due to infection it is perhaps surprising that little attention is paid to infection control during childbirth, especially in low and middle income countries. Apart from the risks of infection during childbirth as a result of unhygienic practices, in India (as well as many other developing countries) there is a growing drive to encourage women to attend health facilities for delivery care [8], with consequent risks for increased levels of morbidity and mortality at these facilities due to overcrowding and poor infection control. With growing concerns related to global infection control and the need to accelerate progress toward MDG 5, urgent action is required to develop and deploy practical interventions against neglected conditions such as puerperal sepsis and other infectious conditions in women.

In circumstances where the increase in the demand for delivery in health facilities is not matched with improvements on the supply side of the health system, such as human resources and drugs, there is a risk of adverse outcomes in terms of complications and deaths. Evidence from historical and contemporary data sets demonstrates puerperal sepsis cases and fatalities as sensitive markers of this demand-supply imbalance [3]. The prevention of infection and of deaths from infection in mothers related to delivery reflects directly many aspects of the quality and safety of obstetric services and thus, in turn, the essential building blocks of the health system – including skills and motivation of health providers, access to drugs and supplies to enable the practise of asepsis and antisepsis.

- 1. **Why sepsis is important in India**

Due to the size of its population, India accounts for 22% (117,000) of the annual maternal deaths worldwide. The estimated maternal mortality ratio in 2005 was 450 deaths per 100,000 [5], a figure which the Indian Government aims to reduce to less than 100 by 2012 [6]. Of the 117,000 maternal deaths each year puerperal sepsis is the second commonest cause after PPH, a recent study in India indicated that sepsis may be responsible for nearly 20% of all maternal deaths [8], if these figures are accurate then PS is responsible for around 23,000 deaths in India every year.

In addition to the role infections play in maternal deaths it is important to remember that infections are also recognised as a major cause of neonatal deaths. A review of studies based in India, South East Asia and the Pacific region which considered hospital based neonatal deaths found that infections were responsible for between 14-36% of deaths. An evaluation of community studies found infection to be responsible for between 8-64% of neonatal deaths [16].

- 1. **Overall plan**

The overall plan for our study is in two phases;

- Phase 1 has already been completed and is described in section 1.4 below
- Phase 2 of the study is the focus for this protocol, where a before and after study will be conducted, with a multi-faceted infection control package introduced as our intervention.
  1. **Work that has already been completed**

The study is part of ongoing work on the subject of Puerperal infection in India. To date three pieces of preliminary work have been completed in the first phase (March 2009-February 2010):

- A literature review which looked at infection control strategies related to delivery care
- A needs assessment in Gujarat state to identify key issues related to infection control in maternity units
- A workshop organised in Gujarat involving the input of government officials, representatives of non-governmental organisations and research groups to help design an intervention

The literature review found that;

- Multifaceted strategies for infection control related to care during delivery (such as organisational and systems changes, feedback, training) are widely recommended.
- Evidence on effectiveness of strategies related to care during delivery is limited by the varied nature and multiple components of different strategies and quasi-experimental designs with no controls.
- There is little specific information related to labour and delivery.
- Evidence on cost-effectiveness in infection control is lacking.

The needs assessment revealed;

- Infection control in maternity units is suboptimal. Although basic supplies and equipment are available, unnecessary and non-evidence based practices were found, protocols and procedures were not available
- Infection control in delivery rooms was poor compared to those in obstetric operation theatres

The workshop was held at the Indian Institute of Management (IIM), Ahmedabad in June 2009 and the proceedings [17] are available via the Immpact website ([www.immpact-international.com](http://www.immpact-international.com/)). The workshop was used to disseminate the information generated by the literature review and the needs assessment, while designing and developing the intervention to be used in the study.

1. **STUDY OBJECTIVES**

Phase 2 of the study will seek to assess whether introduction of a multifaceted strategy identified by our literature review will;

1. Improve maternal infection rates after childbirth
2. Strengthen health systems in hospital settings in India

The underlying primary hypothesis in this study is that, the multifaceted strategy infection control package will result in the formulation and implementation of locally achievable and sustainable action to reduce rates of wound, bloodstream and reproductive tract infections after childbirth. Anticipated distal effects will be improvements in quality of delivery care and strengthening of the health system.

The primary area of interest for our study is nosocomial or hospital acquired infection, which is defined as any infection occurring in women who delivered their baby or the placenta (or both) in the study health facilities. We hope to capture data on women who deliver in hospitals and subsequently contract infections while in hospital (as well as through any obstetric interventions performed).

Many infections during childbirth or after childbirth are acquired in the community but this is not the area of interest for our study. In such a scenario, the source of infection becomes unclear.

1. **DESCRIPTION OF THE INTERVENTION**

As highlighted by our literature review in phase 1 of the study, a multifaceted strategy was identified as the best approach to infection control. Our intervention will have four core infection control elements, and a fifth element of appreciative inquiry as indicated below.

1. Improvement of surveillance systems for infection control
2. Operationalisation of infection control committees
3. Use of an audit, feedback, and problem solving mechanism
4. Development of locally relevant, standardised guidelines and protocols
5. Appreciative inquiry

Appreciative inquiry (AI) is a fairly new and unique concept in infection control, and is the fundamental basis for our intervention. AI being a theory, paradigm of thought and an approach to analysis promotes organisational creativity and learning [7]. Conventionally, organisations tend to look at what is “wrong” in-order to forge solutions for problems, but AI seeks, what is “right” in the organisation in-order to solve problems. AI hypotheses, in-order to solve a problem, the best approach would be;

1. Identification of situations where things worked best
2. Looking at what exactly worked at those times
3. Conceptualising a vision of what we (individuals in the organisation) want in the future
4. Building on what worked best for the future

The concept of Appreciative inquiry is derived from a business model which was originally developed by David Cooperider and Suresh Srivastava as a management technique in the 1980s [25]. Appreciative inquiry seeks to improve perceptions, attitudes and behaviours of individuals as well as improve the standards of care received.

Appreciative inquiry hence brings together groups of people to identify problems and develop solutions, using self-reflective analysis and learning within a supportive environment. Sessions are held to include health facility personnel with diverse roles such as hospital cleaners, ambulance drivers, water engineer, nurses, doctors, administrators etc. Critical events are used in discussions which are non-threatening and non-punitive. Successes and problem-solving are the focus of discussions. In maternal health, it has been implemented at small scale to improve quality of emergency obstetric care in countries such as Bangladesh, India and Nepal. Although its effects were not evaluated formally in these settings, existing evidence suggest benefits of the approach [7].

A diagrammatic representation of the intervention is shown in figure 1. First of all, we will have specified inputs from;

1. Evidence based practice advisor, who will advise us on evidence based practices
2. Local infection control expert, who will guide us on local infection control
3. State technical officer, who will direct us on congruence with the health system e.g. introduction a national accreditation scheme
4. Appreciative inquiry trainer, who will advise us on the AI process.

These four key experts will have inputs on the four standard infection control elements as well as on appreciative inquiry. They will train a group of key people selected from the interventions sites, on the multifaceted package, who will in turn, go back to their facilities and implement it.


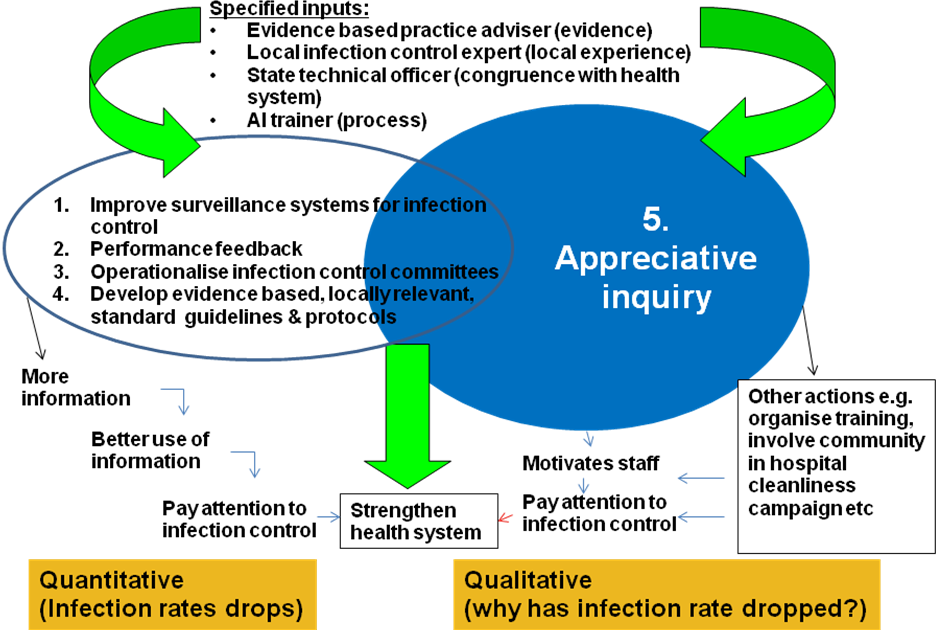


Figure 1: A diagrammatic representation of the multifaceted strategy for infection control

It is hoped from our multifaceted intervention package that two outputs will be seen;

1. Through improvement of surveillance systems, use of performance feedback and functionalising infection control committees, more information on infection control will be generated. This information will then be put into better use e.g. through development of locally relevant guidelines and protocols which in turn, will influence hospital staff to pay attention to infection control. Thus, there will be a decrease in infection rates detected through measurement of quantitative outcomes.
2. We will also discover new actions or interests that will be highlighted by the appreciative inquiry process itself. For example, we may discover training needs for a cleaner, who has never been trained on infection control. The cleaner will then be trained on infection control, which may then motivate him, and in turn lead him to pay attention to infection control. Thus, resulting in a decrease in infection rate. The reason behind this decrease will then be picked up by the qualitative part of our study.

These two outputs will then lead to strengthening of the healthcare system.

1. **RESEARCH QUESTIONS**

The overall research question is:

***What are the effects of introducing a multi-faceted surveillance-audit infection control intervention focusing on childbirth, in hospitals within Gujarat, India?***

Specific research questions can be categorised into two groups:

**Group 1; Infection rates: [quantitative]**

1. What are the levels of puerperal infection amongst women who deliver in hospital?
2. What are the different types of infections that occur?
3. Are infection rates different in different subgroups of women? For example rural-urban residence, rich-poor, different hospitals etc
4. Are infection rates reduced after introduction of the multi-faceted intervention?
5. How are antibiotics used for post natal women [who contract infections]?

**Group 2; Health system and quality of care: [qualitative]**

These questions have two aims;

1. To find out why (if any), there has been a change in infection rates
2. To assess the effect of the intervention on the health system and on quality of care

Specific research questions are;

1. What are the changes resulting from the intervention?
   1. How has the intervention affected the six health service components i.e. service delivery, the health workforce, information, medical products, finance and leadership?
   2. If changes have occurred, are they consistent with the intended goal of reducing infections after childbirth?
   3. Have the changes affected other maternity services like antenatal care, postnatal care, management of other obstetric complications and how have the changes affected them?
   4. Have the changes affected other non-maternity services and how have the changes affected them?
   5. Why did any of the changes described above occur and how was the change achieved?
2. What are the respondents’ perceptions of why the infection rates have changed or not after the intervention?
3. What were the major enablers or barriers encountered, which influenced the achievement or non-achievement of reduction in infection rates?
4. **STUDY DESIGN**

In order to address the two study objectives, the study will have two parts. A quantitative part will aim to establish the number of infections in women delivering in the study hospitals before and after the intervention. A qualitative part will seek to establish whether the intervention leads to improvement in quality of care and strengthening of the health care system.

- 1. **Quantitative Study - Overview**

The quantitative part of the study will begin in July 2010. An initial pilot stage will be carried out for one month. This will inform us on what to expect in the different hospitals i.e. in terms of records kept, number of deliveries etc. Primarily, seven hospitals will be selected which give an expected number of approximately 6000 – 7000 deliveries per year. The rates of deliveries and infections will be monitored, for all deliveries occurring during this period. New mothers will be monitored for any signs or symptoms of infection while in hospital, and also after discharge, where they will be followed up and monitored in the community for 42 days after delivery.

Table 1 shows start and end months’ for each of the stages, and the number of sites involved at each stage.

- Pilot stage – July 2010
- Baseline assessment stage – August to October 2010
- Pre-intervention stage – November to January 2011
- Intervention stage – February 2011 onwards, initially for seven months (may be longer dependant on additional funds)

The baseline assessment stage will be used to enumerate the number of deliveries and post-partum infections in each of the hospitals.[[1]](#footnote-2) This information, along with other characteristics, will be used to allocate the hospitals into two groups such that the different groups of hospitals are matched. These two groups will be randomly allocated either as control or intervention group (see section 5.1.1).

During the Pre-intervention stage, data collection on post partum infections will still continue after baseline stage in the selected study sites.

Table 1: Study timeline

| **Year** | 2010 | | | | | | | 2011 | | | | | | |
| --- | --- | --- | --- | --- | --- | --- | --- | --- | --- | --- | --- | --- | --- | --- |
| **Month** | July | Aug | Sept | Oct | Nov | Dec | Jan | Feb | Mar | Apr | May | June | July | Aug |
| **Stage of study** | Pilot | Baseline assessment stage | | | Pre-intervention stage | | | Intervention stage | | | | | | |
| **No of sites** | Seven Sites | | | | Five Sites | | | | | | | | | |
| **AI training and intervention** |  | | | | AI Training begins in intervention sites | | | Onset of AI intervention | | | | | | |

The length of the intervention will be dependant on additional funds. It is hoped by late March or Early April 2011 there will be enough data available to perform a preliminary analysis on the infection rates before and after the intervention. This data will present a case to the MacArthur Foundation (or any other suitable agencies) requesting for more funds that will facilitate extension of the intervention stage until February 2011. Such an extension will mean there will be an overlap between the data collected in the first two stages, enabling a comparison to be made between the infection rates at the same time of year before and after the intervention. This is important due to possible variation by seasonality (see question in section 9.0). If successful in securing additional funds, the study timeline will be slightly different; with a longer intervention period of 12 months as shown in Table 2.

Table 2: Study timeline with additional funds

| **Year** | 2010 | | | | | | | 2011 | | | | | | | | | | | | |
| --- | --- | --- | --- | --- | --- | --- | --- | --- | --- | --- | --- | --- | --- | --- | --- | --- | --- | --- | --- | --- |
| **Month** | J | A | S | O | N | D | J | F | M | A | M | J | J | A | S | O | N | D | J | F |
| **Stage of study** | Pilot | Baseline assessment stage | | | Pre  intervention stage | | | Intervention Stage | | | | | | | | | | | | |
| **No of sites** | Seven | | | | Five Sites | | | | | | | | | | | | | | | |
| **AI training and intervention** |  |  | | | AI training begins in intervention sites | | | Onset of AI intervention | | | | | | | | | | | | |

- - 1. **Quantitative Study – Site Selection**

Initially seven sites[[2]](#footnote-3) from the central region of Gujarat (see table 1 & 2) will be chosen for the baseline assessment stage. This stage will take three months starting in July. Infections rate will be monitored in these sites, which will be narrowed down to a group of five (but maybe four or six) sites that yield around 6000-7000 deliveries per year. The hospitals will be selected to match as closely as possible a range of criteria including:

- Delivery rates (preference to choose high volume centres)
- Infection rates
- Rates of complications during delivery
  - Instrumental deliveries
  - Caesarean sections
  - Manual removal of placentas
  - Other complications
- Type of catchment area
  - Rural or Urban

During the visit to Ahmedabad in April 2010, preliminary data was obtained listing some of the hospitals with more than 1000 deliveries per year in the local area. Information on; the number of staff, type of staff available at each facility, and the rate of caesarean sections was also obtained.

In addition to the study site criteria discussed above, the other logistical considerations of hospitals are as follows;

- Select hospitals from the Government sector before and after the intervention. If we find NGO hospitals with sufficient deliveries, we will include them in our study.
- Hospitals with more or less the same number of deliveries will be considered. This will give us a larger hospital pool for selection, if we focus on those with 80 to 100 deliveries per month.
- Select hospitals which are easy to put the intervention in place. This further justifies inclusion of NGO hospitals in our study.
- Select hospitals where Post Natal Care (PNC) visits are safe (security wise) and houses are easily accessible. At least 3-5 PNC visits should be possible per day. The nurse (data collector) in the hospital may spend 2 hrs every day filling up forms etc, and then go on PNC visits. She will then go home after completing the PNC visits since she would be staying in the village


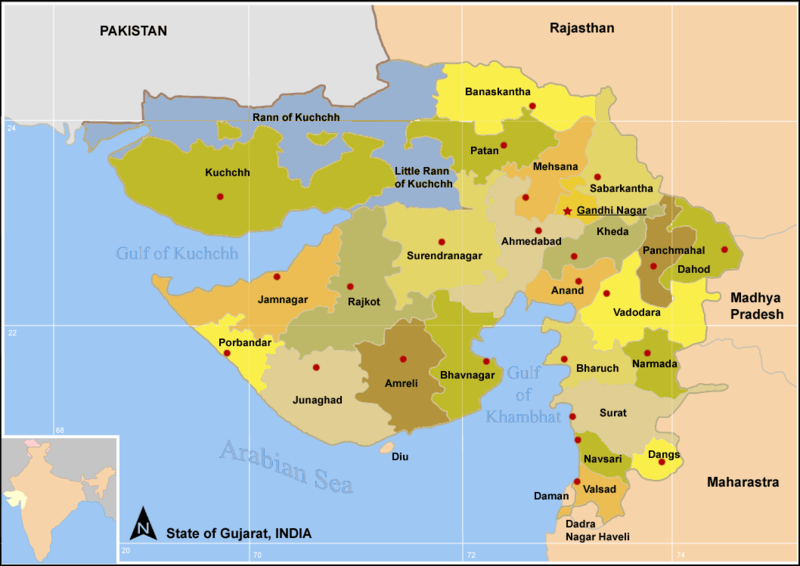


Figure 2: A map of the districts in Gujarat state. The study sites will be selected from those government hospitals in the central region, including Surendranagar, Ahmedabad and Gardinaghar (shown in the diagram as Ghandi Nagar).

- - 1. **Quantitative study – study population**

The study population will be women who delivered in the intervention and control hospitals, who subsequently contract (puerperal) infection of the genital tract.

Women in whom infections of the genital tract are identified after delivery, up to 42 days post partum will be included in the study.

Puerperal infections are defined as those specified in ICD-10 codes 085 and 086 (see annex 1):

- Puerperal sepsis
- Other puerperal infections
- Infection of obstetric surgical wound
- Other infection of genital tract following delivery
- Urinary tract infection following delivery
- Other genital tract infection following delivery
- Pyrexia of unknown origin following delivery
- Other specified puerperal infections

**Inclusion criteria**

- Any woman over 28 week’s gestation who delivers a baby (live or stillborn) in any of the control or intervention hospitals.
- Any woman over 28 weeks gestation who has delivered a baby (in any location be it in the community, a study site or a non-study hospital) who is admitted with the placenta undelivered.

**Exclusion criteria**

- Any woman who delivers a baby (live or stillborn) less than 28 weeks gestation will not be included in the study. This also excludes miscarriage and abortion cases.

Note: Often, these cases are seen in out-patients or admitted in a different ward and are classified as “gynaecologic” cases and not “obstetric” cases

- Any woman admitted to the study site after delivery of the placenta will not be included.

The process for determining whether a case meets the inclusion or exclusion criteria is shown in figure 3.

- - 1. **Quantitative study - Sample size requirements and sampling procedure**

All cases of puerperal infections in women who delivered in any of the study hospitals (be they control or intervention) will be included in the study. Cases of maternal mortality due to puerperal sepsis will be included in the study. However, changes in mortality rates will not be used to measure effectiveness since the study is not of sufficient size to do so. Based on evidence from studies of infection rates in India, we anticipate that infection rates are likely to be at least 10-15% of all hospital deliveries, but possibly may be higher.

Sample size discussed with the statistician: working figure of 6000-7000 women.

- - 1. **Quantitative study – acquiring consent**

Consent should be sought from all eligible women, before assessing whether a woman meets the inclusion criteria or not. The woman will be given the information about the study, and then she’ll read and sign the consent form. If the woman is unable to read, the information will be read to her by the data collector and a thumbprint will be taken in place of a signature. In this instance, the data collector should also sign the consent form, confirming that information on the study has been given. If the woman is incapable of consenting e.g. if she is mentally ill, relatives can consent on her behalf, for the entire study period.

If the woman does not wish to be involved in the study; whether through refusal of consent or for other reasons, the data collector should fill a non-participation form (NPF) for all these women. (NPF, annex 2, See explanation in section 5.1.6).


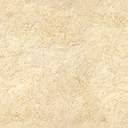


No

Yes

No

No

No

No

No

No

No

No

No

Yes

Yes

Yes

Yes

Yes

Yes

Yes

Yes

Yes

Yes

Has this woman delivered a baby (live or stillborn) in the past 24 hours?

Was this baby greater than 28 weeks gestation?

Exclude

Were both the baby and the placenta delivered in the same hospital?

Was that hospital one of the study sites?

Include

Were the different hospitals both control hospitals?

Were the different hospitals both intervention hospitals?

Was the baby delivered in the community and the placenta delivered in hospital

Were both the baby and the placenta delivered in the community?

Exclude

Was the baby delivered in a control hospital and the placenta delivered in an intervention hospital?

Was the baby delivered in a non-study hospital and the placenta delivered in a study hospital?

Include

Include as a delivery in the intervention hospital

Was the baby delivered in an intervention hospital and the placenta delivered in a control hospital?

Include as a delivery in the control hospital

Include as a delivery in the hospital that the placenta was delivered in

**Figure 3: Inclusion and exclusion criteria**

- - 1. **Quantitative study – data collection and case identification in the hospital**

**Data collection:** A data collector will be assigned to each study site. He/ she will visit labour and post natal wards every morning, having the following tasks to fulfil.

- Establish how many deliveries occurred within 24 hours since their last visit.
- Establish which women have been discharged in the previous 24 hours since their last visit.
- Identify mothers who have given birth in the previous 24 hours, seek informed consent, check that they meet the inclusion criteria and then administer part 1 and 2 of the case identification form. If appropriate, the data collector should complete part 3 of the case identification form that depends on the responses of part 2.
- Administer part 2 of the case identification form to previously recruited women recovering in the ward on daily basis.

When the data collector (DC) arrives at the maternity ward each morning he or she should first identify the number of women present, and then identify women who have delivered in the past 24 hours. After establishing the number of new deliveries, the DC should consult the delivery register to confirm the numbers. If there are more deliveries listed than there are new mothers on the ward, then this could indicate that a woman has delivered in the hospital since the DC’s last visit and was discharged before the DC’s arrived the next morning. In this example the woman will have been ‘missed’ by the study. If this happens, then the DC should fill out the non-participation form (NPF) in annex 2 and state the reasons why the woman was discharged so soon after delivering.

The completion of NPFs for each birth not included in the study, will allow the study team to accurately record the number of births observed during the study period. Thus, the data can be presented in relation to included births, effectively giving numerators as well as denominators. When writing up the study, comments on the number of births included as a proportion of all the births during that period can be made. For example, “6489 births were included in the study, which was 92.1% of all the births in the study sites during the study period. Of those that were not included, 3.8% of women did not meet the inclusion criteria, 2.1% of women did not consent to take part and 1.0% of women were lost to follow up”.

This is clearly illustrated in figure 4


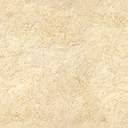


**A-Number of women admitted into maternity unit/ department**

**C**-Number of women delivered baby or placenta or both in the health facility

**D**-Number of women who consent to participating in the study

**B**-Number of women who delivered both baby and placenta at home or outside the study health facility who are admitted to maternity unit for other reasons e.g. postpartum haemorrhage, sepsis or pre-eclampsia

**F**-Number of women consistently followed up during the study

**E**-Number of women who do not consent to participate in the study

Complete the NPF form

**G**-Number of women lost to follow up (~≤1% expected)

**H-Total number of women who participated in the study from beginning till the end**

Exclude

Include

Figure 4: Study population

**Note:** Women could be admitted to maternity unit or department for a variety of reasons e.g. for postpartum haemorrhage, sepsis, post partum eclampsia etc

**Case identification:** The case identification form will be used to identify cases for the study. It is comprised of three parts:

**Part 1:** contains the consent and contact details forms’. The consent form is simple and clear which is only signed (by the woman and DC) after the study has been explained to the woman. The contacts form records the woman’s details such as her name, age, the date and time of delivery, her address etc (see in the quantitative data collection instrument).

There is also space for detailed directions to her address if she lives in a remote or difficult area to locate. Women should also be asked if they are likely to be living anywhere else for the 42 days post-delivery, as most women may spend time after childbirth with a relative. If this is likely then that address should be recorded in section B of this part. The DC should enquire about the next of kin, who the follow up team will contact if they have trouble locating the mother during follow up or post natal visits. This section will record the woman’s phone number that will be used for follow up. If the woman doesn’t own or have access to a telephone, it should be clearly noted. If possible this part of the case identification form should be filled out within 24 hours after child birth.

**Part 2** is comprised of a list of screening questions that identify whether the woman is likely to have an infection or not. Women will be screened on admission to rule out infections obtained outside the hospital. After which, women who have delivered and are included in the study will also be screened using the same set of questions.

Part 2 contains two sections:

***Section A****;* comprising of signs and symptoms questions as below;

- Are you hot/ sweaty?
- Have you experienced any nausea/vomiting
- Have you experienced any pain in the pelvis, abdomen, perineal area, perineal or caesarean section wound
- Have you experienced any discomfort during urination
- Have you experienced any offensive vaginal discharge
- Do you feel you have a raised temperature
- If you had an episiotomy or caesarean section wound, is it red, swollen or opening

***Section B****;* comprising of antibiotics use questions;

This section will serve to highlight issues around indiscriminate antibiotic use, which may be a pointer, to low infection rates. It could be a possible confounder.

If the woman answers yes to any questions in section B, the data collector should proceed to part three of the form after completing section B. If the woman answers no to all of the screening questions in part two, then there is no need for further questions that day.

**Part 3** of the form includes much more detailed questions about the woman’s signs and symptoms that are consistent with infection. Answers to questions in part 3 will be used to decide whether the woman has an infection or not and what type of infection it is.

For each day a woman is on the maternity ward post delivery and prior to discharge, she would be asked screening questions in part two. Every time the woman answers yes to any of section A signs and symptoms questions, the DC should insert a new part three form, which she’ll complete.

When a woman is discharged from the ward, the DC should appropriately note the date in part one and two of the case identification tool.

The procedure for assessing women in hospital is also explained in the flow diagram in figure 3.

- - 1. **Quantitative study – case identification and data collection in the community**

Women delivering in any of the study sites will be followed up in the community for 42 days post partum. The community follow up will be done in several ways;

- Using a self reported symptom card with drawings alongside each question
- Contacting the women by phone on days 7, 14 and 42,
- If necessary, visiting women in their homes.

The self reported symptom card will be issued to every woman recruited into the study on Day 1 post partum, and the DC will explain what it is and how to use it.

The symptom card has a list of screening questions similar as part 2 signs and symptoms section of the case identification form. Adjacent each symptom, there are 42 boxes which correspond to the 42 days a woman will be followed-up after delivery. The woman should tick or cross appropriately for each of the signs and symptoms (See figure 4 and an example illustrated in the annex). If the woman ticks any of the symptoms, she will be asked to contact the data collector by phone on a number indicated on the symptom card. The DC will then arrange to visit the woman in her home where he/she will complete part 3 of the case identification form.


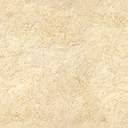


No

No

No

No

No

Yes

Yes

Yes

Yes

Yes

Have any women been discharged since yesterday?

Check the patient’s notes and check with staff on the ward to ensure that the woman has been discharged.

Check and record the women present on the ward compared to those present yesterday.

Add the date of discharge into part one of that woman’s case identification form and follow that woman up with a phone call on the 7^th^, 14^th^ and 42^nd^ days post partum.

Counting the women on the ward and using the delivery register, have any women delivered since yesterday?

Administer part two of the case identification form. Does the woman answer yes to any of the questions in part two?

Complete the NPF in annex 2 and finish

Is the woman happy to take part in the study?

Complete a consent form. Does the woman meet the inclusion criteria?

Complete parts one and two of the case identification form. Does the woman answer yes to any of the questions in part two?

Complete part three of the case identification form for that woman and finish

Finish

Are there any women recovering on the maternity ward who have already been recruited into the study during previous visits?

Yes

Was the woman recruited in the study?

No

Yes

No

Yes

Complete the NPF & finish

No

**Figure 4:** Beginning in the top left, this is a flow diagram explaining how women are identified in hospital and how the case identification form is administered.

The DC will also contact the mothers on days 7, 14 and 42 post partum through telephone calls and or visits. During these visits the DCs will make sure that the women are filling the forms appropriately. They will administer the screening questions in part two and, if required, the more detailed questions in part three will shortly ensue.

|  | **On admission** | **Date of delivery** | **1** | **2** | **3** | **4** | **5** | **6** | **7*** | **8** | **9** | **10** | **11** | **12** | **13** | **14*** |
| --- | --- | --- | --- | --- | --- | --- | --- | --- | --- | --- | --- | --- | --- | --- | --- | --- |
| **Tick the number of days post partum** | **N/A** |  |  |  |  |  |  |  |  |  |  |  |  |  |  |  |
| **Does the woman complain of any of the following symptoms? Tick the symptoms as they appear on each postpartum day.** | | | | | | | | | | | | | | | | |
| 1. **Hot/sweaty** |  |  |  |  |  |  |  |  |  |  |  |  |  |  |  |  |
| 1. **Nausea/vomiting** |  |  |  |  |  |  |  |  |  |  |  |  |  |  |  |  |
| 1. **Pain in pelvis, abdomen, perineal area or wound** |  |  |  |  |  |  |  |  |  |  |  |  |  |  |  |  |
| 1. **Discomfort during urination** |  |  |  |  |  |  |  |  |  |  |  |  |  |  |  |  |
| 1. **Offensive vaginal discharge** |  |  |  |  |  |  |  |  |  |  |  |  |  |  |  |  |
| 1. **Raised temperature** |  |  |  |  |  |  |  |  |  |  |  |  |  |  |  |  |
| 1. **Wound redness, swelling or opening** |  |  |  |  |  |  |  |  |  |  |  |  |  |  |  |  |
| 1. **None of the above** |  |  |  |  |  |  |  |  |  |  |  |  |  |  |  |  |
| 1. **Are you using any antibiotics? If yes, complete section B (antibiotics questions). If no, leave out section B** |  |  |  |  |  |  |  |  |  |  |  |  |  |  |  |  |
| 1. **Tick day of discharge** |  |  |  |  |  |  |  |  |  |  |  |  |  |  |  |  |
| 1. **Tick day of referral**   **State which hospital__________________** |  |  |  |  |  |  |  |  |  |  |  |  |  |  |  |  |
| 1. **Tick day of death** |  |  |  |  |  |  |  |  |  |  |  |  |  |  |  |  |

**Figure 4:** An example of the symptom card issued to mothers at the beginning of the study. The days after delivery are listed in the top row. Asterisks* on days 7 and 14 indicate when follow up calls or visits will be made by the data collector.

**Note**. On the actual card the days will run till day 42 and the data collector contact number indicated at the back of the card.

- - 1. **Quantitative study – outcome measures and study variables**

The main quantitative outcome measures will be maternal mortality and morbidities resulting from puerperal infections including bloodstream, reproductive tract and wound infections. This will be disaggregated by type of infection. Explanatory variables include:

- Place of delivery (by hospital)
- Type of attendant involved in care during childbirth (midwife, doctor, no one) (i.e. of the women with infections, do certain types of infection happen by type of delivery attendant?)
- Mode of delivery (e.g. vaginal, instrumental, caesarean) and/or type of obstetric intervention (e.g. manual removal of placenta, suturing of perineal tear)
- Duration of hospital stay
- Residence of woman
- Socio-economic status or caste
- Antibiotic use; start and end dates (Ante-partum, Intra-partum and postpartum)
  - 1. **Quantitative study – verification and analysis**

Supervisors will collect data from the study sites on a monthly basis and fill out the supervision checklist (see section 6.2). He/she will also count the number of deliveries in the delivery register to see if the number tallies with the number of case identification forms and non-participation forms. This data will be brought back to IIM for entry into a software package familiar to the data entry personnel such as Excel, Access, Epi-Info or SPSS. Some indicators will be generated every month and others on a less frequent basis. During data entry, the data will be verified by allocating different types of infections to individuals who display particular combinations of signs and symptoms, using the guide in annex 2

Table 3, 4 and 5 lists the indicators identified for the study. Examples of data tables with key indicators are provided below.

Note: The seven study hospitals will fall either in intervention group or control group, but not both.

Table 3

| **Monthly incidence of puerperal infections in women**  **=** number of infections in a month X 100%  total births in that month | | | | | | | | | | | | | | | | |
| --- | --- | --- | --- | --- | --- | --- | --- | --- | --- | --- | --- | --- | --- | --- | --- | --- |
|  | Intervention Group | | | | | | | | Control Group | | | | | | | |
|  | before | | | | after | | | | Before | | | | after | | | |
| Month | 1 | 2 | 3 | etc | 7 | 8 | 9 | etc | 1 | 2 | 3 | etc | 7 | 8 | 9 | etc |
| Hospital 1 |  |  |  |  |  |  |  |  |  |  |  |  |  |  |  |  |
| Hospital 2 |  |  |  |  |  |  |  |  |  |  |  |  |  |  |  |  |
| Hospital 3 |  |  |  |  |  |  |  |  |  |  |  |  |  |  |  |  |
| Hospital 4 |  |  |  |  |  |  |  |  |  |  |  |  |  |  |  |  |
| Hospital 5 |  |  |  |  |  |  |  |  |  |  |  |  |  |  |  |  |
| Hospital 6 |  |  |  |  |  |  |  |  |  |  |  |  |  |  |  |  |
| Hospital 7 |  |  |  |  |  |  |  |  |  |  |  |  |  |  |  |  |
| Total Incidence rate (%) |  |  |  |  |  |  |  |  |  |  |  |  |  |  |  |  |

**Note:** Point prevalence will be determined independently for all the seven hospitals whether they fall in the intervention group or control group as described in the format below (Table 4).

Table 4

| **Point prevalence of different infections each postnatal day, e.g.**  **=** no. of puerperal sepsis cases occurring on a specific day post delivery X 100%  Total number of puerperal infections found | | | | |
| --- | --- | --- | --- | --- |
|  | Intervention Group | | Control Group | |
|  | Before | After | Before | After |
| Puerperal sepsis day 1-5 |  |  |  |  |
| Puerperal sepsis day 6-10 |  |  |  |  |
| CS wound infection day 1-5 |  |  |  |  |
| CS wound infection day 6-10 etc |  |  |  |  |

- 1. **Qualitative Study – Overview**

There are two aims for the qualitative part of the study;

- To measure the effect of the intervention on the health system, by considering the six different dimensions of the health system, according to the WHO definition (See annexe 3).
- To measure the effect of the intervention on the quality of care

This part of the study will involve in-depth interviews of key informants who will be carefully selected from a set framework, with knowledge and understanding of different aspects of the health care system.

- - 1. **Qualitative study – participant selection**

As stated above, it is important to select different participants who will have a good understanding of different aspects of the health system. To ensure this is possible, participants from a range of different professions and vocations within the hospitals will be selected. Table 5 shows participants at the state level and the hospital level who will be included.

| **Position** | **Likely numbers of individuals holding the position** | **Approximate numbers we aim to interview** |
| --- | --- | --- |
| State level health officer | 1 | 1 |
| State level QA officer | 1 | 1 |
| District level medical officer | 1 | 1 |
| Hospital superintendant | 1 | 1 |
| Nurse/midwife in charge of maternity units | 3-4 | 1 |
| Nurse in charge of medical/surgical wards | 12 | 1 or 2 |
| Pharmacists | 2-3 | 1 |
| Doctors | 2-5 | 1 |
| Nurse midwives | 20 | 1 |
| Nurse auxiliaries | 20 | 1 |
| Cleaners | 5 | 1 |

Table 5: Key Informants to be interviewed

Note: Individuals in the grey boxes will be interviewed in every study hospital, whereas only one State level health officer, one State level QA officer and one District level medical officer will be interviewed since these individuals cover the whole of Gujarat state.

- - 1. **Qualitative study – Interview timing**

As explained in part 5.1 the length of the study will initially be for 13 months including baseline stage with the possibility of an extension of five more months if extra funds are secured early in 2011. We will only conduct 1 key informant interview after the intervention stage **(**more details can be found in a separate document; the qualitative study protocol JH (V. 29^th^ July 10) – **using infection control as an entry point for improving the quality of delivery care and strengthening health systems in developing countries.)**

Table 6: KIIs timing for 13 months

| **Year** | 2010 | | | | | | | 2011 | | | | | | |
| --- | --- | --- | --- | --- | --- | --- | --- | --- | --- | --- | --- | --- | --- | --- |
| **Month** | July | Aug | Sept | Oct | Nov | Dec | Jan | Feb | Mar | Apr | May | June | July | Aug |
| **Stage of study** | Pilot | Baseline assessment stage | | | Pre-intervention Stage | | | Intervention stage | | | | | | |
| **No of sites** | Seven Sites | | | | +/- Five Sites | | | | | | | | | |
| **AI training and intervention** |  | | | | AI Training begins in intervention sites | | | Onset of AI intervention | | | | | | |
| **KI Interviews** |  |  |  |  |  |  |  |  |  |  |  |  |  | 1^st^ |

Table 7: KIIs timing for 18months

| **Year** | 2010 | | | | | | | 2011 | | | | | | | | | | | | | | | |
| --- | --- | --- | --- | --- | --- | --- | --- | --- | --- | --- | --- | --- | --- | --- | --- | --- | --- | --- | --- | --- | --- | --- | --- |
| **Month** | J | A | S | O | N | D | J | F | M | A | M | J | J | A | S | O | N | | D | | J | | F |
| **Stage of study** | Pilot | Baseline assessment stage | | | Pre-intervention stage | | | Intervention Stage | | | | | | | | | | | | | | | |
| **No of sites** | Seven | | | | +/- Five Sites | | | | | | | | | | | | | | | | | | |
| **AI training and intervention** |  | | | | AI training begins in intervention sites | | | Onset of AI intervention | | | | | | | | | | | | | | | |
| **KI Interviews** |  |  |  |  |  |  |  |  |  |  |  |  |  |  |  |  |  |  | |  | |  | |

The key informant interview will give an impression of the status of the healthcare system in relation to how it supports obstetric infection control. The interview will show what kind of effect the intervention is having on the health care system and also highlight what kind of changes have occurred since the intervention was put into place. Most importantly, the KII will seek to clarify the reasons behind these changes, and whether or not the intervention is responsible for these changes.

The KIIs will be able to identify if there are any other changes that may have occurred in the health system, outside the intervention, which may have affected infection control practices in the hospitals. For example, introduction of an accreditation scheme within the last month by the hospital head. This scheme may require the facility to implement infection control practices for it to be accredited. As a result of the scheme, infection rates in the hospitals will begin to drop. In such instances, the KII will be able to pick such changes.

- - 1. **Qualitative study – Interview topic guide**

The topic guide for the KII is given in a separate document; the qualitative study protocol JH (V. 29^th^ July 10) – **using infection control as an entry point for improving the quality of delivery care and strengthening health systems in developing countries.** The topic guide is arranged into sections which reflect different dimensions of the health system, and questions to guide the interviewer. These questions ask about different indicators which help establish the relative strength of the health system, and strength of the different dimensions of the health system.

Although the topic guide is quite long it is assumed that only some of the questions will be appropriate for each key informant (KI). For example a nurse may know much more about service delivery than governance, and so be able to answer many more of the service delivery questions. Similarly, the state health officer will probably know much more about governance and health system financing but less about medical products. In light of this, it is recommended that the interviewer would ask the same questions to each KI. However, the interviewer should be aware of the level of knowledge for the various individuals on different dimensions of the healthcare system.

- - 1. **Qualitative study – Interviews and consent forms**

Interviews will be conducted by a research fellow from IIM, preferably in a quiet and private place where interruptions will be unlikely. Before visiting the site, the interviewer should contact the individual in advance to arrange a convenient time to meet, as well as provide information regarding the study and what the interview will involve.

At the time of the interview, the interviewer should find out if the interviewee is happy to participate in the study. If so, consent will be sought, this will be confirmed by a signature from the interviewee. (See consent form: annex 7).

It may be helpful to explain to the interviewee that there are no right or wrong answers, with everything they say being confidential and will not be shared with anyone outside the study team. It is important that they understand this, as it is likely to affect how open they are during the interview, hence, how good the quality of data will be.

Open ended questions will be used in the interview to probe for further information, exercising caution not to lead the interviewee. Each question has sub-questions which gives the interviewer an indication of the expected information.

- - 1. **Qualitative study – outcome measures and study variables**

The qualitative component will explore the research questions related to the health system and quality of care. The main outcomes of interest are related to the research questions and will describe:

- Actions and practices that have resulted from the intervention, in relation to care during delivery but also in other maternity care, especially postnatal care within and outside the hospital setting, e.g. attitudes and perceptions of health personnel, managers and decision makers, frequency and effectiveness of postnatal visits in the home, cleaning of instruments, hand washing practices
- Perceptions of key informants (health personnel) and service users relating to the six health system components of;
- Service Delivery (e.g. Quality, type)
- Health Workforce (e.g. Availability, workload, motivation)
- Information (e.g. Sources, type, appropriateness, quality)
- Medical products, Vaccines and Technologies (e.g. Availability, quality of drugs, equipment, supplies)
- Financing (e.g. Sufficiency of resources, flows)
- Leadership and Governance (e.g. Who are the leaders key to introducing change, how do they function, gaps)

Explanatory variables cannot be specified at this point, and will emerge as the qualitative data is collected and analysed, but examples might include:

- Barriers and enabling factors encountered which influenced the achievement or non-achievement of reduction in infection rates
- Reasons why changes in any of the six health system components might have occurred after the intervention was introduced.
  - 1. **Qualitative study – data analysis**

After recording, the interviews will be transcribed, coded and thematically analysed. Appropriate statistical software such as Nvivo or N6 will be employed. During analysis the data will be arranged into various themes according to the six healthcare components mentioned in 5.2.5

1. **Data collection instruments for both quantitative and qualitative parts**

There will be three data collection instruments used in the study i.e. case identification form, woman’s self reporting guide and topic guide for KII. Table 8 shows how the data collection instruments relate to the research questions and indicators used.

Included in this section is also a brief explanation of the Supervision checklist (6.2). (Although it is not one of the data collection instruments, highlighting it will ensure the data collected and recorded is of good quality).

Table 8: Data collection in relation to research questions and indicators

| **Research question** | **Indicators** | **Related tasks** | **Data source/instrument used** |
| --- | --- | --- | --- |
| What are the levels of puerperal infection amongst women who deliver in hospital? | - Monthly incidence of puerperal infections in women - Monthly incidence of associated neonatal infection | Collect monthly data on   - live births - all births   in each hospital | Labour room and CS theatre registers (or hospital statistics if these are reliable) and supervision checklist |
|  |  | Hospital staff/ANM in community complete a case identification form for any woman suspected of having an infection who has delivered in the hospital | Case identification form |
| What are the different types of infection that occur? | - Point prevalence of different infections each postnatal day - Incidence of different types of infection | Completion of case identification form | Case identification form |
| Are infection rates different in subgroups of women? | Infection rates in:   - village/town dwelling women - women of different castes - women delivering in the 2 different hospitals | Completion of case identification form | Case identification form |
| Are infection rates reduced after the intervention is introduced? | - Overall incidence of puerperal infection before and after intervention and in control site - Overall incidence of neonatal infection | Completion of case identification form | Case identification form |
| Health system and quality of care questions | See section 5.2 | Key informant interviews | Topic guide |

- 1. **Case identification form**

This will be used to identify women with infections for the quantitative part of the study. The case identification form will be split up into three parts:

**Part one**

- Consent statement
- Contact information

**Part two**

- Screening questions
- Antibiotic questions

**Part three**

- In-depth questions about symptoms and signs

After the case identification forms are collected, the answers given in part three will be used to determine whether the woman had an infection or not.

- 1. **Supervision Checklist**

One of the Research Fellows from IIM will be appointed as a supervisor and they will visit and collect the case identification and non participation forms in each of the study sites fortnightly, for the first two to three months then the visits will be reduced to monthly for the rest of the period. While collecting the various forms they will also count the number of deliveries in the labour register to ensure that all the births have been counted and recorded either with a case identification form or a non participation form. The supervisor will also inspect the forms completed to check that they are being filled out properly by the data collectors.

- 1. **Women’s self reporting card**

This data collection instrument has already been described in section 5.1.5 and there is an example of it in the annex. Data collectors should give this to the woman during their first meeting together and take care to explain the importance of filling it in accurately every day. To demonstrate how it should be filled, the DC should fill it in with the mother during the first two or three days that she is in hospital.

ASHAs (community health and social care workers) should also be made aware of the self reporting cards so they too can remind women to fill them during their routine visits during the post-natal period.

As an incentive to encourage the mothers to keep the form safe and fill it in every day, a small reward will be given to those mothers that have successfully completed the form when they are collected in during the last visit from the DC on the 42^nd^ day post partum.

- 1. **Key informant topic guide**

A topic guide is provided for each of the KII which should be used to guide the interview. Below is a list of points that interviewers may find helpful to remember while conducting the KII. Some of the points refer to the example transcripts in Box 1.

- Try to ask open, non-leading questions that avoid encouraging the interviewee to answer in a particular way, for example rather than asking “is the hospital overcrowded?”, instead ask, “How does the size of the hospital compare to the number of people who use it?”
- Encourage the KI to elaborate on points that are of interest to the study, for example in transcript two in box 1 the Interviewer uses open phrases such as “tell me more about what has happened since the hospital employed an infection control nurse”. This elicits much more information than the question, “and because of that, has infection rates reduced?”
- Be prepared to follow up on points that may be of interest to the overall research aims and objectives, even if they are not on the topic guide. The topic guide should be just that – a guide, and interviewers should explore points that come up during interview as long as they are relevant to the overall research questions. An example of this is given in transcript two in Box 1; the KI implies that there have recently been new standards and policies adopted and the interviewer goes on to ask more about that in the next question.
- As much as possible the interview should flow like a normal conversation, instead of becoming a list of questions and answers. It is hoped that the interviews will describe what is happening in the hospital and why, since the intervention was introduced (or not). KIs will only do this if they are encouraged to speak freely about their own thoughts and experiences.
- More information about conducting qualitative interviews can be found in the references given below.[[3]](#footnote-4)

Box 1: Example transcript giving two slightly different conversations

**Transcript One**

***KI***: “There has been an improvement since the hospital employed an infection control nurse”

***Interviewer***: “And because of that, have infection rates reduced?”

***KI***: “Yes, I think so”

**Transcript Two**

***KI***: “There has been an improvement since the hospital employed an infection control nurse”

***Interviewer***: “tell me more about what has happened since the hospital employed an infection control nurse”

***KI***: “Well, there is someone always around who has a responsibility to ensure that the new hospital policies are adhered to, she serves as something of a reminder to everyone. She is also someone to go to with any problems we may have, if we can’t meet the new standards and policies. For example we were always running out of bleach before and so the floors weren’t being cleaned properly, however when she was told about this she asked management staff in the hospital to change their ordering system, and now we hardly ever run out of anything. Because she is always around she is easier to ask about that sort of thing, because down on the wards we hardly ever see the hospital manager, and even if we did we wouldn’t talk to him about ordering more bleach.”

***Interviewer***: “So the hospital has new standards and policies? Can you tell me some more about them?”

***KI***: “After we started having those meetings last January it became clear that different people were doing very different things, so it was decided that some written standards should be produced so everyone new what they should do and when they should do it, with regard to cleaning the delivery room….”

**ETHICAL CONSIDERATIONS AND ETHICS**

Ethical permission will be sought in India, according to standard procedures. Ethical issues important in this project include:

- Ensuring informed consent of the participants is obtained by simple yet comprehensive explanation of the study and by recording consent provided through signatures, thumbprints or formal documentation of verbal consent.
- Ensuring patients and staff in the study are not losing out unduly due to benefits of the intervention in one site and not another e.g. by identifying a study ‘guardian’ [10]
- Maintaining confidentiality of information, anonymity and sensitivity especially in relation to personal medical details and in situations such as the death of a woman or newborn (where the interests of the woman, family and health workers may need to be considered)
- Ensuring adequate care is provided when cases of infection are identified.
- Roll out of intervention to control sites and recommended to non study sites if intervention is found to be effective
- Use of local resources and knowledge whenever possible

**Ethics**

- Inclusion of women who had still births or neonatal deaths. We will include these women in our study to avoid profound bias. Our data collectors will be trained appropriately to ensure sensitivity in handling these women is exercised.
- If our study picks up an infection in a woman that the clinician may have missed; the clinician will be informed.
- All our study hospitals will be given a clear outline of the study and its objectives. They will also be told of the benefits of falling into control or intervention groups, as well as the basis for selection of each cluster i.e. randomised. Both groups will benefit from our study as shown in table 9.

Table 9: Benefits in both control & intervention groups

| **Control** | **Intervention** |
| --- | --- |
| - **Able to know the levels of infections among women delivering in the hospital** | - **Able to know the levels of infections among women delivering in the hospital** |
| - **Able to know the different types of infections that occur** | - **Able to know the different types of infections that occur** |
| - **Able to know whether the infection rates occur among different sub-groups of women** | - **Able to know whether the infection rates occur among different sub-groups of women** |
| - **Able to know antibiotic use ante-partum, intra-partum and post-partum** | - **Able to know antibiotic use ante-partum, intra-partum and post-partum** |
| - **Overall, there will be less work load i.e. data collection processes** | - **Able to know whether the infection rates reduced after introduction of the multi-component intervention** |
|  | - **Overall, there will be more work load i.e. data collection processes due to the intervention** |

- Calling the woman and asking sensitive questions on phone. The person calling will not be a stranger. It will be our data collectors. But the big question is; “***Does our caller identify themselves on phone if the person answering the call is not our participant e.g. husband, relatives etc***?” This is a sensitive issue, as probably the woman doesn’t want the husband, relatives or neighbours to know her health issues. We will address it by inserting a question in the consent form asking the woman if she is happy for the caller to identify herself.
- The women will also be asked when it is most appropriate to call; this is to ensure that her privacy is respected.

**8.0 Potential biases for both quantitative and qualitative parts of the study**

| **Possible bias** | **Means to minimise bias** |
| --- | --- |
| Selected hospitals not comparable | - Matched by identified criteria as far as possible |
| Other (non research) intervention introduced which affects results | - Key informant interviews will identify |
| Puerperal infections (especially minor ones) missed because initial screening (i.e. which cases have a case identification form completed) poorly done by data collectors | - Could complete a case identification form for all women seen post delivery, but this is time consuming |
| Puerperal infections missed because case identification form is poorly completed | - Training of data collectors |
| Incorrect estimates of denominator data (undercounting or over counting by double counting) | - Identify most accurate source of data - Compare and cross check different data sources - Every delivery in each study site will be counted via either the case identification form or the non-participation form |
| Women delivering a baby in one study hospital and the placenta in another may be counted twice | - It is likely to be a very rare event. But if it happens, e.g. If a woman comes to a study site via referral from a different hospital, this will be noted on the case identification form that has a question in Part 1; section B, asking where does this woman come from? Home/ referral etc providing further details. We will only consider delivery of the placenta in our study site in this instance. |
| Loosing women to follow up | - Data collectors will take contact information from the woman, as well as her next of kin. Detailed instructions will be sought to locate her house. Several telephone calls will be attempted to both the woman and the next of kin before a visit will be made to all of the addresses recorded. |

| Women may not fill in their self reported card | - Data collectors will take care to explain the importance of the card when they give it to women during their first meeting after delivery. - Data collectors will fill the card in with the women while they are in hospital awaiting discharge - Data collectors will contact the women on days 7, 14 and 42 to check that they are still filling in the self reporting card - Women will receive a gift if they have completed the form correctly when it is picked up by the ASHA on the 42^nd^ day post partum. |
| --- | --- |
| Women may fill in their self reporting card but fail to call the data collector | - The number will be a free number (query it’s likelihood) so it will not cost the women anything to call the data collector if they begin to develop any of the symptoms listed on the self reporting card. |
| Wrong key informants selected | - Wide consultation in selected study site to ensure local knowledge used to identify appropriate key informants |
| Interviews with key informants do not elicit required information | - Ensure interviewer skilled in interviewing, and understands requirements of research and research questions |
| Failure to recruit sufficient numbers |  |
| Telephone bias to women who are rich and literate | - After our initial ground work in April 2010, we found that at least 95% of the woman access to a phone |
| The Infection status of women on arrival to hospital |  |
| Women who are not able to read and write [literacy bias] | - Use of drawings in the women’s self reported cards |

**9.0 QUESTIONS ABOUT THE STUDY**

***Why did the study change from having one intervention hospital and one control hospital to including several hospitals?***

In order for the study to be adequately powered each site (control and intervention) need around 3000 deliveries per year. There were only two hospitals in Gujarat state that have around 3000 deliveries per year and these were not appropriately matched for the number of deliveries or for other criteria. It was decided instead, as a compromise, that a group of control hospitals and a group of intervention hospitals could be used. Each group would contain two or three hospitals to give an overall number of deliveries of around 3000 per year, per group. Since there are many more hospitals to choose from that have around 1000 deliveries per year, it would be much more straightforward to match these hospitals for other criteria as well.

***Why is the number of hospitals decreasing after the first 3 months, why not just keep the same number of hospitals from start to finish?***

During the visit to Ahmedabad in April 2010 it became clear that information on infections were not routinely recorded and so there was no way of finding out what the infection rates were in each of the hospitals. It was also suggested that the information on delivery complications may be unreliable. Since it is important to match hospitals according to the baseline delivery rates and levels of infection, it was decided to carry out a baseline assessment stage. This would provide data for three months on the number of deliveries in each of the hospitals and the number of infections in each of the hospitals. This information would then be used to accurately match five or six of the seven original sites.

***Why is the baseline stage not running for 12 months, this way the natural seasonality of infections would not affect the study?***

Ideally the baseline assessment stage and the pre-intervention stage combined would run for 12 months before the beginning of the intervention stage, such that seasonality of birth and infection could be accounted for. However, given the increased number of sites involved and so the increased number of data collectors, the budget will only allow for a 3 month baseline assessment stage and a 3 month pre-intervention stage.

The monsoon season in Gujarat runs between July and August. It is hoped that by at least including this period within the pilot and baseline assessment stage, it will give an indication of any seasonality of infections during the whole study period.

***Why not start with more hospitals than seven in the study, to get a range of different hospitals to choose from, which would allow more accurate matching?***

Ideally it would be good to include as many hospitals as possible in the baseline assessment stage, however the more sites included and the more data collectors employed, the more expensive the study becomes.

***What is the point of the KIIs, why not just do an inventory or inspection to assess the strength of the health system?***

As described in the protocol, there are two aims for Key Informant Interviews;

- To assess the strength of the health system – before and after the intervention.
- To understand any changes that may have occurred, the reasons behind the changes, and whether or not they were due to the intervention or any other changes that may have taken place.

**9.0 DETAILED TIMELINE APRIL-DEC 2010**

*Note: Intervention will commence 1^st^ January 2011.

| **Month 2010** | **Apr** | | | | **May** | | | | **Jun** | | | | **Jul** | | | | **Aug** | | | | **Sep** | | | | **Oct** | | | | **Nov** | | | | **Dec** | | | |
| --- | --- | --- | --- | --- | --- | --- | --- | --- | --- | --- | --- | --- | --- | --- | --- | --- | --- | --- | --- | --- | --- | --- | --- | --- | --- | --- | --- | --- | --- | --- | --- | --- | --- | --- | --- | --- |
| **Week** | 1 | 2 | 3 | 4 | 1 | 2 | 3 | 4 | 1 | 2 | 3 | 4 | 1 | 2 | 3 | 4 | 1 | 2 | 3 | 4 | 1 | 2 | 3 | 4 | 1 | 2 | 3 | 4 | 1 | 2 | 3 | 4 | 1 | 2 | 3 | 4 |
| Field test instruments | X |  |  |  |  |  |  |  |  |  |  |  |  |  |  |  |  |  |  |  |  |  |  |  |  |  |  |  |  |  |  |  |  |  |  |  |
| Finalise instruments |  | X |  |  |  |  |  |  |  |  |  |  |  |  |  |  |  |  |  |  |  |  |  |  |  |  |  |  |  |  |  |  |  |  |  |  |
| Select study sites | X | X |  |  |  |  |  |  |  |  |  |  |  |  |  |  |  |  |  |  |  |  |  |  |  |  |  |  |  |  |  |  |  |  |  |  |
| Finalise ethical approval | X | X |  |  |  |  |  |  |  |  |  |  |  |  |  |  |  |  |  |  |  |  |  |  |  |  |  |  |  |  |  |  |  |  |  |  |
| Identify data collectors |  |  |  |  |  |  | X | X |  |  |  |  |  |  |  |  |  |  |  |  |  |  |  |  |  |  |  |  |  |  |  |  |  |  |  |  |
| Train data collectors |  |  |  |  |  |  |  |  | X | X |  |  |  |  |  |  |  |  |  |  |  |  |  |  |  |  |  |  |  |  |  |  |  |  |  |  |
| Start data collection |  |  |  |  |  |  |  |  |  |  |  |  | X |  |  |  |  |  |  |  |  |  |  |  |  |  |  |  |  |  |  |  |  |  |  |  |
| IIM RF/data collection supervisor does supervision visits and collect case identification forms from hospitals and from community |  |  |  |  |  |  |  |  |  |  |  |  | X |  | X |  | X |  | X |  | X |  |  |  | X |  |  |  | X |  |  |  | X |  |  |  |
| Data entry and analysis for quantitative indicators |  |  |  |  |  |  |  |  |  |  |  |  | X | X |  |  | X | X |  |  | X | X |  |  | X | X |  |  | X | X |  |  | X | X |  |  |
| Share findings with Aberdeen |  |  |  |  |  |  |  |  |  |  |  |  |  |  | X |  |  |  | X |  |  |  | X |  |  |  | X |  |  |  | X |  |  |  | X |  |
| Identify appropriate key informants |  |  |  |  |  |  |  |  |  |  |  |  |  |  |  |  |  |  |  |  |  |  |  |  |  |  |  |  |  |  |  |  |  |  |  |  |
| First key informant interviews* |  |  |  |  |  |  |  |  |  |  |  |  |  |  |  |  |  |  |  |  |  |  |  |  |  |  |  |  |  |  |  |  |  |  |  |  |
| Preliminary summary and analysis of qualitative data |  |  |  |  |  |  |  |  |  |  |  |  |  |  |  |  |  |  |  |  |  |  |  |  |  |  |  |  |  |  |  |  |  |  |  |  |
| Share findings with Aberdeen |  |  |  |  |  |  |  |  |  |  |  |  |  |  |  |  |  |  |  |  |  |  |  |  |  |  |  |  |  |  |  |  |  |  |  |  |
| Start preparations for intervention and appreciative enquiry training |  |  |  |  |  |  |  |  |  |  |  |  |  |  |  |  |  |  |  |  |  |  |  |  |  |  |  |  |  |  |  |  |  |  |  |  |
| Call planning meeting |  |  |  |  |  |  |  |  |  |  |  |  |  |  |  |  |  |  |  |  |  |  |  |  |  |  |  |  |  |  |  |  |  |  |  |  |

**ANNEX 1 – ICD-10 codes for post partum infections**

| **O85** |  | **Puerperal sepsis** | |
| --- | --- | --- | --- |
|  |  | Puerperal:  · endometritis  · fever  · peritonitis  · septicaemia | |
|  |  | Use additional code (B95-B97), if desired, to identify infectious agent. | |
|  |  | ***Excludes:*** | obstetric pyaemic and septic embolism ( [O88.3](http://apps.who.int/classifications/apps/icd/icd10online/go85.htm#o883%23o883) )  septicaemia during labour ( [O75.3](http://apps.who.int/classifications/apps/icd/icd10online/go60.htm#o753) ) |

| **O86** |  | **Other puerperal infections** | | | |
| --- | --- | --- | --- | --- | --- |
|  |  | Use additional code (B95-B97), if desired, to identify infectious agent. | | | |
|  |  | ***Excludes:*** | infection during labour ( [O75.3](http://apps.who.int/classifications/apps/icd/icd10online/go60.htm#o753) ) | | |
| **O86.1** |  | **Infection of obstetric surgical wound** | | | |
|  |  | Infected: | | | |
|  |  | · Caesarean section wound  · Perineal repair | | }  } | following delivery |
| **O86.2** |  | **Other infections of genital tract following delivery** | | | |
|  |  | Cervicitis  Vaginitis | | }  } | following delivery |
| **O86.3** |  | **Urinary tract infection following delivery** | | | |
|  |  | Conditions in N10-N12, N15.-, N30.-, N34.-, N39.0 following delivery | | | |
| **O86.4** |  | **Other genitourinary tract infections following delivery** | | | |
|  |  | Puerperal genitourinary tract infection NOS | | | |
| **O86.5** |  | **Pyrexia of unknown origin following delivery** | | | |
|  |  | Puerperal:  · infection NOS  · pyrexia NOS | | | |
|  |  | ***Excludes:*** | puerperal fever ( [O85](http://apps.who.int/classifications/apps/icd/icd10online/go85.htm#o85%23o85) )  pyrexia during labour ( [O75.2](http://apps.who.int/classifications/apps/icd/icd10online/go60.htm#o752) ) | | |
| **O86.6** |  | **Other specified puerperal infections** | | | |

**ANNEX 2: Sources of definitions for symptomatic case identification**

ICD-10 [12] was used to help decide which infections to include as post partum infections, the section ‘complications predominantly related to the puerperium (O85-O92)’ was reviewed and the subsections which described infections were selected, these were:

- O85 – Puerperal sepsis
- O86 – Other puerperal infections

**Puerperal Sepsis**

Different sources may give slightly different diagnostic criteria for puerperal sepsis [10, 11, 12]. A WHO Technical Working Group on puerperal sepsis convened in 1993 and suggested the following definition [9, 13] for use in developing countries which will be used in this study, as follows.

Infection of the genital tract occurring at any time between the onset of the rupture of membranes or labour and the 42^nd^ day postpartum in which two or more of the following are present;

- 1. Pelvic pain
  2. Fever i.e. oral temperature 38.5^o^C/101.3^o^F or higher on any occasion
  3. Abnormal vaginal discharge, e.g. presence of pus
  4. Abnormal smell/foul odour of discharge and
  5. Delay in the rate of reduction of size of the uterus (<2cm a day for the first 8 days)

**Other puerperal infections**

A textbook of obstetrics [18] as well as recent review on vaginitis and cervicitis [19] were used to define the symptoms of peritonitis and genital tract infections. The textbook [18] was used again to define the criteria for UTIs, while two reviews [20,21] and information from the textbook were used to define the diagnostic criteria for caesarean section wound infections and episiotomy/ perineal tear wound infections.

The case identification form for maternal infections is given in the appendix. The first part of the form aims to identify cases of puerperal sepsis as defined by the WHO technical working group [9].

***Peritonitis***

1. Does the patient have;
   1. Rebound tenderness[[4]](#footnote-5)
   2. Nausea or vomiting
   3. Abdominal pain, or pelvic pain, or tenderness of the abdomen on palpation

***Genital tract infection*** – definition according to ref [20]

1. Does the patient have[[5]](#footnote-6);
   1. Pelvic pain
   2. abdominal pain
   3. A delay in the reduction of the size of the uterus (<2cm a day for the first 8 days)
2. Purulent lochia or malodourous lochia

***Urinary Tract Infection*** – definition according to ref [18]

1. Symptoms must only have developed post partum
2. Pain on micturition[[6]](#footnote-7)
3. Cloudy or discoloured urine
4. At least one of the following;
   1. Increased frequency
   2. Urgency
   3. Hesitancy
   4. Dribbling
   5. Purulent urethral discharge

***Episiotomy wound or perineal tear wound infection*** – using [18] and [20]

1. An Episiotomy or perineal tear was acquired during the patient’s most recent delivery[[7]](#footnote-8)
2. With regard to episiotomy or perineal tear wound
   1. Is there discharge from the wound
   2. If yes, is the discharge purulent
   3. Has the wound begun to open up
3. With regard to the episiotomy or perineal tear wound;
   1. Is there bruising around the wound
   2. Is there redness around the wound
   3. Is there swelling around the wound
   4. Is there area around the wound tender

***Caesarean section (CS) wound infection***

1. Did the mother deliver her most recent baby by caesarean section[[8]](#footnote-9)
2. With regard to the CS wound;
   1. Has the wound begun to open
   2. Is there a purulent discharge
3. With regard to the CS wound;
   1. Is there bruising around it
   2. Is area around it red
   3. Is there swelling around the wound
   4. Is the area around the wound tender

**ANNEX 3: BACKGROUND ON HEALTH SYSTEMS**

The aims of the qualitative part of this study are;

1. To find out why (if any), there has been a change in infection rates
2. To assess the effect of the intervention on the health system and on quality of care

Data will be collected on aspects of the health system in order to gain insight into the following research questions:

1. What are the changes resulting from the intervention?
   1. How has the intervention affected the six health service components; service delivery, the health workforce, information, medical products, finance and leadership?
   2. If changes have occurred, are they consistent with the intended goal of reducing infections after childbirth?
   3. Have the changes affected other maternity services like antenatal care, postnatal care, management of other obstetric complications and how have the changes affected them?
   4. Have the changes affected other non-maternity services and how have the changes affected them?
   5. Why did any of the changes described above occur and how was the change achieved?
2. What are the respondents’ perceptions of why the infection rates have changed or not after the intervention?
3. What were the major enablers or barriers encountered, which influenced the achievement or non-achievement of reduction in infection rates?

We have used the WHO toolkit for health system strengthening <http://www.who.int/healthinfo/statistics/toolkit_hss/en/index.html> as the main reference for the development of the topic guide.

A “health system consists of all organisations, people and actions whose primary intent is to promote, restore or maintain health” [1]

Key Informant in-depth interviews will be used to assess the relative strength of the health system using the 6 individual components of a health system, outlined by WHO [2].

**Service Delivery**

Good health services are those that deliver effective, safe, quality personal and non personal health interventions to those that need them, when and where needed, with minimum waste of resources [2]. Service Delivery can be broken down into five parts;

1. **Service Availability:** This is measured by the number and distribution of health facilities per 10,000 population and the number and distribution of in-patient beds per 10,000 population.
2. **Service Capacity (General)**: This is the proportion of health facilities that meet basic service capacity standards. The WHO toolkit goes on to divide service capacity into five parts with several questions for each which assesses the basic capacity of a facility, these are given in box one.
3. **Service Capacity (Specific):** It is the number and distribution of health facilities with basic service capacity per 10,000 population. This relates to the service capacity of a facility that provides key services such as malaria control or control of sexually transmitted diseases. In terms of safe motherhood facilities claiming to offer basic delivery care would be expected to meet certain criteria, whereas those offering emergency obstetric care would be expected to meet a different set of criteria (see box 2).
4. **Service Utilisation:** it is calculated and measured by the number of outpatient department visits per 10,000 per year
5. **Service Quality:** Health care services must be safe, effective, patient centred and timely.

Box 2. Core items found in facility to ensure a basic level of service capacity [3]

| 1. Basic amenities |
| --- |
| 1. Regular water supply from safe source on-site (or within 500m) 2. Sanitary facilities: client latrine 3. Waiting area protected from sun and rain 4. Communication equipment (landline or mobile phone or short wave radio) 5. Electricity: routinely available during service hours or a backup generator with fuel |
| 2. Basic equipment |
| 1. Adult weighing scale 2. Child weighing scale 3. Thermometer 4. Stethoscope 5. BP cuff 6. Refrigerator 7. Needles and syringes |
| 3. Infection control |
| 1. Functioning sterilization equipment with power source for method: 2. autoclave, dry heat sterilization, boiling and steaming, chemical disinfection (chlorine base or glutaraldehyde solution) 3. Written guidelines or protocols 4. Sharps container or box 5. Soap, disinfecting solution, gloves, and water (in service delivery area) |
| 4. Human resources |
| 1. Minimum staffing requirement according to national guidelines (qualified staff) 2. Proportion of health workers present on the day of assessment |
| 5. Tracer drugs and diagnostics |
| 1. Availability tracer drugs: the proportion of the tracer medicines that are present and non-expired on the day of the assessment 2. Capacity to obtain basic laboratory results within one day: Hb; HIV test, syphilis test and malaria blood test if appropriate. |

Box 3. Criteria for assessing service capacity in those facilities offering basic delivery care and emergency obstetric care

| Level of Delivery care | Staff and Training | Equipment | Treatment/ Prevention |
| --- | --- | --- | --- |
| Basic delivery care | - Guidelines - Trained service provider (midwife) | - Emergency transport - Gloves - Delivery Bed - Partograph - Examination light - Scissors, blade, cord clamp and suction apparatus - Needles and syringes - IV solution with infusion set - Suture material and needle holder | - Antibiotic eye ointment for newborn - Skin disinfectant - Injectable oxytoxic/ ergometrine - Oral antibiotic - Anticonvulsant - Magnesium sulphate or valium (injectable) - Injectable antibiotic |
| Emergency obstetric care | - Guidelines - Trained service provider | - Vacuum extractor - PAC – Vacuum aspirator - D&C kit - Blood transfusion - Caesarean Section - Respiratory support unit (infant sized ambu- bag) | - As above |

**Health Workforce or Human Resources for health**

Health workforce is defined as “all people engaged in actions whose primary intent is to enhance health”. This definition includes both clinical and management staff, such as nurses, midwives, administrators, and support staff (such as accountants and porters) [3]. If good performance is to work in ways that are responsive, efficient and fair, then available resources and circumstances should be taken into consideration. For example, a sufficient number, mix (gender; male or female) and distribution of staff that are competent, responsive and productive can produce good performance [2].

The health workforce comprises of suitably qualified personnel, who are well distributed, and proportionate to the population served by the facility. There should be a good mix of male and female staff. For example in some cultures, lack of female staff may act as a barrier to utilisation of health services by women.

There are several indicators recommended by the WHO toolkit [3] that can be used to assess human resources such as;

- - Number of health workers per 10,000 population
  - Distribution of health workers by occupation/specialisation, region place of work and sex
  - Annual number of graduates of health professions educational institutions per 100,000 population, by level and field of education

**Health information Systems**

A well functioning health information system is one that ensures the production, analysis, dissemination and use of timely and reliable information on health determinants, health systems performance and health status [2]. Health information systems have four main functions;

- - Data generation
  - Compilation
  - Analysis and synthesis
  - Communication and use

Although they are sometimes used synonymously, the health information system has a broader remit besides monitoring and evaluation. It often provides alerts and early warning capabilities, support patient and health facility management, enable planning, support and stimulate research, permit health situation and trends analysis, support global reporting and underpin communication of health challenges to diverse users [3].

Indicators used for measuring Health Information Systems [3] are;

1. **Health surveys**

- Country have a 10 year cost survey plan that covers all priority health topics and takes into account other relevant data source
- Two or more data points available for child mortality in the past 5 years
- Two or more population-based data points for maternal mortality in the last 10 years
- Two or more data points for coverage of key health interventions in the last 5 years
- One or more data point on smoking and adult nutritional status in the last 5 years

1. **Birth and death registration**

- Percentage of births registered
- Percentage of deaths registered
- ICD10 used in district hospitals and causes of death reported to national level

1. **Census**

- Census completed within past 10 years
- Population projections for districts and smaller administrative areas available in print and electronically, well documented

1. **Health facility reporting**

- Number of institutional deliveries available, by district, and published within 12 months of the preceding year
- HIV prevalence for relevant surveillance populations published within 12 months of preceding year
- Country website for health statistics with latest report and data available to the general public
- Reporting of notifiable diseases makes use of modern communication technology and reporting of statistics from district to national levels is web-based
- Percentage of districts that submit timely, complete, accurate reports to national level
- Data quality assessments carried out and published within last 3 years
- International Health Regulations implemented according to international standards

1. **Health system resource tracking**

- At least one national health accounts completed in last 5 years
- National database with public and private sector health facilities, that are Geo-coded should be available and updated within last 3 years
- National database with health workers by district and main cadres updated within last 2 years
- Annual data on availability of tracer medicines and commodities in public and private health facilities

1. **Capacity for analysis, synthesis and validation of health data**

- There is a designated and functioning institutional mechanisms charged with analysis of health statistics, synthesis of data from different sources and validation of data from population and facility sources
- There is a national set of indicators with targets and annual reporting to inform annual health sector reviews and other planning cycles
- There is a national micro-data archive for health surveys and census that is operational
- Survey data are used to assess and adjust routine reports from health facility on vaccinations with the results published within 12 months of the preceding year
- A burden of disease study has been conducted within the last 5 years by national stakeholders
- A study of health systems performance has been carried out within the last 5 years by national stakeholders
- There is national commitment to transparency in data dissemination and acknowledgement of uncertainty
- The official annual health statistics report has been published within 12 months of the preceding (calendar or fiscal) year

**Medical Products, Vaccines and Technologies**

A well functioning health system will ensure equitable access to essential medical products, vaccines and technologies of assured quality, safety, efficacy and cost-effectiveness and their scientifically sound and cost-effective use [2].

Besides accessibility and availability of medical products, it is important that the manufacturing process is of quality standards, the suppliers are reliable, and the medical products are stored, prescribed and administered appropriately.

There are two main indicators that can be used;

- Percent of facilities that have all tracer medicines and commodities in stock i.e. on the day of visit, and in the last three months.
- Ratio of median local medicine price to international reference price (median price ratio or MPR) for core list of drugs [3].

**Measurement of access to drugs: different components and sub-indicators [3]**

**STRUCTURE:**

1. **Access to essential medicines/technologies as part of the fulfilment of the right to health, recognized in the constitution or national legislation (MTSP country progress indicator). Target: Yes**
2. **Existence and year of last update of a published national medicines policy (1). Target: Yes, and updated within the last ten years**
3. **Existence and year of last update of a published national list of essential medicines (1) (MTSP indicator). Target: Yes, and updated within the last two years**
4. **Legal provisions to allow/encourage generic substitution in the private sector (1). Target: Yes**

**PROCESS**

1. **Public and private per capita expenditure on medicines (1, 2). Target: country specific $ value**
2. **Percentage of population covered by health insurance (1, 2). Target: country specific; ultimately 100%**
3. **Average availability of 30 selected essential medicines in public and private health facilities (3) (MTSP country progress indicator). MTSP target: 80%; probably needs country-specific targets**
4. **Median consumer price ratio of 30 selected essential medicines in public and private health facilities (3) (MTSP country progress indicator). MTSP target: below 4x world market reference price**
5. **Percentage mark-up between manufacturers' and consumer price (3). Target: country specific, but generally below 70% (target to be refined)**

**Explanatory notes:**

1. **Standard WHO pharmaceutical survey indicator, collected every 4 years (last in 2007) from most countries**
2. **Standard information, available from National Health Accounts, routinely collected from a large number of countries**
3. **Standard WHO/HAI indicator from national medicine pricing surveys, currently available from 45 countries (2007)**

**Health Systems Financing**

Health system financing is defined by WHO as the function of health system concerned with mobilization, accumulation and allocation of money to cover the health needs of the people, individually and collectively in the health system. The purpose of health financing is to make funding available, as well as to set the right financial incentives to providers. It also ensures that all individuals have access to effective public and personal health care, and are protected from financial catastrophe or impoverishment associated with having to pay for health services [2].

It is important that a health system is adequately funded such that it can deliver appropriate level of services. The healthcare system should also ensure that the way it raises funds does not expose its users to the risk of impoverishment through paying for healthcare.

The financing system can be divided into three parts

- - Revenue collection
  - Fund pooling
  - Purchasing/provision of services

***Revenue collection*** describes the way people pay directly for the service, direct user charges for example. In this case everyone pays the same fee for a service so the rich pay the same as the poor, this can deter people from using care and push people below the poverty line.

***Fund pooling*** describes the way people may pay into a pool while they are healthy, from which funds are drawn when they are ill, these pooled funds may come from tax or health insurance contributions.

***Purchasing and provision of services*** considers how the money is spent. Money should be spent efficiently with the minimum of waste; there should also be ways of guarding against corruption.

**The objectives and actions of Health System Financing can be broken down into;**

1. Raising funds for health - In low income countries this must come from external and internal sources. More and more reliable external funds are needed in most countries but more can be done to raise funds or to raise them more efficiently.
2. Improvement of financial risk protection and coverage for vulnerable groups – In most countries this requires moving away from direct out of pocket payments and towards a form of prepayment with risk pooling – tax or insurance based
3. Improvement in the efficiency of resource utilisation
4. Improved financial transparency and management at operational levels

**There are various indicators used such as;**

1. Data on total health expenditures routinely collected and reported
2. Patient/household out of pocket expenditures of accessing or obtaining services collected intermittently
3. In countries with widespread health insurance: Number (%) of people/households covered by health insurance by population group and specifically for poor vulnerable groups
4. Information on Government expenditures on wages and salaries readily available
5. Availability of data on government expenditure on priority problems, by level of government.
6. Number and percentage of facilities meeting established national financial management criteria.

**Health Systems Governance**

Leadership and governance involves ensuring strategic policy frameworks exist and are combined with effective oversight, coalition building, the provision of appropriate regulations and incentives, attention to system-design and accountability [2]. In particular the relationships between different stakeholders should be managed such that the health system is accountable. Accountability is made up of five different aspects;

1. Delegation of services or an understanding of how services will be supplied
2. Financing to ensure that adequate services are available to deliver services
3. Performance of the actual supply of services to ensure that services are delivered to the best standard
4. Receipt of relevant information to evaluate or monitor performance
5. Enforcement such that, according to performance, sanctions may be imposed or rewards made.

**Indicators used for Governance;**

1. Health worker absenteeism in public health facilities
2. Proportion of government funds which reach district level facilities
3. Stock-out rates (absence) of essential drugs in health facilities
4. Proportion of informal payments within the public health care system
5. Proportion of pharmaceutical sales that consist of counterfeit drugs
6. Existence of effective civil society organisations in countries with mechanisms in place for citizens to express views to government bodies

**References**

1. World Health Report 2000 Health Systems: Improving Performance. WHO Geneva
2. Everybody’s Business: strengthening health systems to improve health outcomes: WHO’s framework for action. WHO Geneva, 2007.
3. Toolkit for monitoring health systems and strengthening (draft version). Accessed 16^th^ March 2010 <http://www.who.int/healthinfo/statistics/toolkit_hss/en/index.html>
4. WHO service availability mapping questionnaire. Accessed 17^th^ March <http://www.who.int/healthinfo/systems/samquestionnaires/en/index.html>
5. A guide to rapid assessment of human resources for health. Accessed 19^th^ March <http://www.who.int/hrh/tools/en/Rapid_Assessment_guide.pdf>

**ANNEX 4: TIMELINE (FROM FUNDED PROPOSAL)**

Original historical timeline (updated timeline is provided in the protocol)

|  | Year 1 | | | | Year 2 | | | | Year 3 | |
| --- | --- | --- | --- | --- | --- | --- | --- | --- | --- | --- |
|  | Oct ‘09-Mar ‘10 | | Apr ‘10-Sep ‘10 | | Oct ‘10-Mar ‘11 | | Apr ‘11-  Sep ‘11 | | Oct ‘11-Mar ‘12 | Apr ‘12  Sep ‘12 |
| **“AN INFECTION CONTROL INTERVENTION STUDY”** | | | | | | | | | | |
| **Objective 1: To conduct interrupted time series study of an infection control intervention package in India.** | | | | | | | | | | |
| Work up of an operational protocol comprising site selection criteria, sampling and data management procedures, analysis plans, ethical approvals | XXXXX | | |  | |  | |  |  |  |
| Set up of intervention package within existing systems |  | | |  | | X | |  |  |  |
| Data collection in pre and post intervention stages |  | | | XXXXX | | XXXXX | | XXXXX | XXX |  |
| Analysis and reporting |  | | |  | |  | |  | XX | XXXXX |
| ***Objective 3: To implement an engagement strategy to communicate the findings and lessons from the studies and enhance their influence on policy and practice.*** | | | | | | | | | | |
| Identify sources for additional or co-funding of this and future proposals on obstetric infection control | | XXXXX | |  | | XXXXX | |  | XXXXX |  |
| Liaise with national and international stakeholders | | XXXXX | | XXXXX | | XXXXX | | XXXXX | XXXXX | XXXXX |
| Use websites and e-communication modalities of collaborating partners and interest groups to post progress reports, findings of study and policy briefs | |  | | X | | X | | X | X | X |
| Publish research papers and policy briefs on study findings | |  | |  | |  | |  | XXXXX | XXXXX |

**ANNEX 5: References**

1. Hill K et al. Estimates of maternal mortality worldwide between 1990 and 2005: an assessment of available data. Lancet 2007; 370: 1311-19
2. WHO 2008 Measuring health systems strengthening and trends: a toolkit for countries. June 2008. Geneva: WHO
3. De Brouwere V, Tonglet R, Van Lerberghe W. 1998 Strategies for reducing maternal mortality in developing countries: what can we learn from history of western countries? Tropical Medicine and International Health, 3: 771-782
4. *The Global Burden of Disease: 2004 Update.* Geneva: World Health Organization, 2008
5. UNICEF – India Statistics. Available at;

<http://www.unicef.org/infobycountry/india_statistics.html>

1. International Institute for Population Sciences (IIPS) and Macro International. 2007. National Family Health Survey (NFHS-3), 2005–06: India: Volume I. Mumbai: IIPS. Available at; <http://www.measuredhs.com/pubs/pub_details.cfm?ID=723&ctry_id=57&SrchTp=ctry&flag=sur&cn=India>
2. CA Carter,MC Ruhe,S Weyer,D Litaker,RE Fry,KC Stange An appreciative inquiry approach to practice improvement and transformative change in health care settings. Quality management in health care 16: 194-204
3. SK Battacharyya, AK Majhi, SL Seal, S Mukhopadhyay, G Kamilya and J Mukherji. Maternal mortality in India: A 20-year study from a large referral medical college hospital, West Bengal. Journal of Obstetrics and Gynaecology Research (2008) Vol 34, No. 4 499-503
4. WHO (1996) The prevention and management of puerperal sepsis. Report of technical working group. WHO/FHE/MSM/95.4
5. Wager GP et al 1980 Puerperal infectious morbidity: relationship to route of delivery and antepartum *Chalmydia trachomatis* infection. American Journal of Obstetrics and Gynaecology 138(7 pt2): 1028-1033
6. WHO (1994) Maternal Health and Safe Motherhood Programme Division of Family Health. Mother-baby Package: Implementing Safe Motherhood in Countries. WHO/FHE/MSM/94.11
7. International Classification of Diseases and related Health Problems, 10^th^ Revision. Geneva, Switzerland: WHO, 1992 (WHO/FHE/MSM//95.4)
8. Abouzahr C, Aaahman E, Guidotti R. Puerperal sepsis and other puerperal infections. In: Murray CJL, Lopez AD, eds. Health Dirmensions of Sex and Reproduction: The Global Burden of Sexually Transmitted Diseases, Maternal Conditions, Perinatal Disorders and Congenital Abnormalities. Geneva, Switzerland: WHO; 1998
9. Yokoe DS, Christiansen RJ, Sands KE, Livingston J, Shtatland ES and Platt R. Epidemiology of and Surveillance for Postpartum Infections. Emerging Infectious Diseases (2001) Vol. 7 No. 5: 837-841
10. Dolea C and Stein C. Global burden of maternal sepsis in the year 2000. (2000) WHO
11. Stoll BJ. The Global Impact of Neonatal Infection. Clinics in Pernatology (1997) Vol 24. No. 1 pp1-21
12. Immpact “Using Infection Control as an Entry Point for Improving the Quality of Delivery Care and Strengthening Health Systems in Developing Countries” A workshop jointly organised by the Indian Institute of Management, Ahmedabad and Immpact at the University of Aberdeen, UK 2-4 June 2009 Available from the June 2009 News Archive
13. Gabbe SG, Neibyl JR, Simpson JL (Eds) Obstetrics: normal and problem pregnancies. 4^th^ Edn. Churchill Livingston.
14. Laine C, Williams S and Wilson JF (2009) In the clinic: Vaginitis and Cervicitis. Annals of Internal Medicine Vol 151 No 5 ITC3-1
15. Tharpe N. (2008) Postpregnancy genital tract and wound infections. Journal of Midwifery and Womens Health 53;236-246
16. Burke C. (2009) Perinatal Sepsis. Journal of Perinatal and Neonatal Nursing. 23(1);42-51
17. Bang AT, Reddy HM, Baitule SB, Deshmukh MD, Bang RA (2005) The Incidence of Morbidities in a cohort of neonates in rural Gadchiroli, India: Seasonal and Temporal Variation and a Hypothesis About Prevention. Journal of Perinatology 25:S18-S28
18. Department of Health (2007) Taking Blood Cultures: A summary of best practice
19. Thompson and Madeo (2009) Blood Cultures: Towards zero false positives. Journal of Infection Prevention Vol 10 Supp. 1 s24-26.
20. Cooperrider, D., Srivastva. “Appreciative Inquiry In Organisational Life”. W. Pasmore & R. Woodman (Eds), *research in organisational change and development*, 1987. Vol 1; 129-169

**References used in instrument development**

1. Tharpe N. (2008) Postpregnancy genital tract and wound infections. Journal of Midwifery and Womens Health 53;236-246
2. Burke C. (2009) Perinatal Sepsis. Journal of Perinatal and Neonatal Nursing. 23(1);42-51
3. Subbe CP et al (2001) Validation of a modified early warning score in medical admissions. Q J Medicine 94;521-526
4. Gabbe SG, Neibyl JR, Simpson JL (Eds) Obstetrics: normal and problem pregnancies. 4^th^ Edn. Churchill Livingston.
5. Bang AT, Reddy HM, Baitule SB, Deshmukh MD, Bang RA (2005) The Incidence of Morbidities in a cohort of neonates in rural Gadchiroli, India: Seasonal and Temporal Variation and a Hypothesis About Prevention. Journal of Perinatology 25:S18-S28
6. Toolkit for monitoring health systems and strengthening (draft version). Accessed 16^th^ March 2010 <http://www.who.int/healthinfo/statistics/toolkit_hss/en/index.html>
7. WHO service availability mapping questionnaire. Accessed 17^th^ March <http://www.who.int/healthinfo/systems/samquestionnaires/en/index.html>
8. A guide to rapid assessment of human resources for health. Accessed 19^th^ March <http://www.who.int/hrh/tools/en/Rapid_Assessment_guide.pdf>

**Annex 6: Quantitative data collection instruments**

**QUANTITATIVE**

**DATA COLLECTION INSTRUMENTS**

- case identification form
- women’s self reporting card

Version 29th July 2010

| CASE IDENTIFICATION NUMBER |  |  |  |  |
| --- | --- | --- | --- | --- |

**Annexe 6a – Case identification form**

**Case Identification Form**

| CASE IDENTIFICATION NUMBER |  |  |  |  |
| --- | --- | --- | --- | --- |

**Instructions for quantitative data collection**

1. This is a before-after control study. The study is interested in nosocomial (hospital acquired) puerperal infections.
2. All women who deliver in hospital study sites will be screened. The initial screening will identify if an infection is likely, and if it is, a form will be completed investigating the symptoms in more detail.
3. In addition, all women who deliver in hospital study sites will be followed up after their discharge from hospital for 42 days post partum by phone (on days 7, 14 and 42) and /or with a visit to her home if indicated during the phone call.
4. Women who deliver in the community are not included in the study (see operational protocol for more details)
5. This booklet is in three parts:

**Part 1**: comprises of;

Section A: Consent form

Section B: Non-participation form (NPF)

Section C: Contact details form.

**Part 2**: comprises of screening questions;

Section A: Signs and symptoms of infection

Section B: Antibiotic use

**Part 3**: comprises of in-depth questions to be completed, only if the woman reports any of the signs and symptoms in part 2.

1. Women with puerperal infections will be identified through:
   1. Screening of cases admitted in hospital
   2. Screening of cases after discharge (by phone)
   3. Identification of cases in hospital (using case identification form)
   4. Identification of cases during community follow up visits (using case identification form)
   5. Hand held card
2. A unique case identification number should be assigned to each woman so that their case forms can be linked during data analysis. Please complete the case ID number on each page of all the forms, including the hand held card.
3. If any infection is picked by our study and may have been missed by the clinician, the clinician should be informed immediately.

| CASE IDENTIFICATION NUMBER |  |  |  |  |
| --- | --- | --- | --- | --- |

**Part 1**

**Section 1A:** Consent form

- **The consent form should be filled out for all women who have delivered in the study hospitals.**
- **Ensure you explain the reason of the study, and obtain the woman’s informed consent.**
- **Explain the need for follow up by phone as some personal questions may be asked on phone.**

| Data collector’s name |  |
| --- | --- |
| Has the study been explained to the woman and her consent to participate obtained? | (A) Yes |
|  | (B) No(ensure that woman is briefed on the study) |
| Are you happy when we call you, we reveal our identity? E.g. If phone belongs to husband, relative etc | (A) Yes |
|  | (B) No |
| “I declare that the study has been explained to me and I willingly participate in this study”  Obtain signature of woman  Date:.......................................... | “I declare that I have fully explained the study to the woman and she has verbally indicated that she is willing to participate in the study”  Signature of data collector on behalf of the woman (if woman not literate or if phone call)  Date:.......................................... |

| CASE IDENTIFICATION NUMBER |  |  |  |  |
| --- | --- | --- | --- | --- |

**Section 1B:** Non-participation form

**Fill in this form if a woman delivers a baby (live or still born) or placenta in one of the study sites, but she (for whatever reason) is not included in the study. At the end of the study this will allow enumeration of the number of women included in the study, as a proportion of the number of women who gave birth in the study sites during that period.**

| 1. Today’s date |  | | |
| --- | --- | --- | --- |
| 1. Date woman delivered |  | | |
| 1. Name of study site |  | | |
| 1. This woman was not included in the study for the following reason(s). Please tick as appropriate. | | | |
| 1. The woman did not give consent | |  |  |
| 1. The woman did not fulfil the inclusion criteria | |  | If yes, please use the space below to explain why  …………………………………………….. |
| 1. The woman delivered and was then discharged from the maternity ward in between visits from the data collector | |  | If yes, please use the space below to explain why the mother discharged herself so soon after delivery.  …………………………………………….. |
| 1. The woman delivered and was transferred from the study site to a different hospital within 24 hours | |  | If yes, please use the space below to explain why the woman was transferred. |
| 1. None of the above | |  | Please give details of the any other reason below.  ……………………………………………  …………………………………………… |

| CASE IDENTIFICATION NUMBER |  |  |  |  |
| --- | --- | --- | --- | --- |

**Section 1C:** Contact details

- **This section should be filled out for all women who give birth in the study hospitals.**
- **When collecting address details, make sure you have the correct address (es) and phone number(s) of how the woman can be reached in the first 42 days after delivery.**

| 1. First name |  | |
| --- | --- | --- |
| 1. Husband’s name |  | |
| 1. Own family name |  | |
| 1. Age of woman | ……………………………………………. | |
| 1. Date of admission to hospital |  | |
| 1. Where did this woman come from? | (A) Home | |
|  | (B) Referral | |
|  | (C) Other. Please specify below  ……………………………………………. | |
| 1. Name of hospital where delivered (baby or placenta) | ……………………………………………. | |
| 1. What was delivered | (A) Baby | |
|  | (B) Placenta | |
|  | (C) Both Baby and Placenta | |
| 1. Who delivered conducted delivery of the baby/ placenta | (A) Doctor | (D) Dai |
|  | (B) Midwife | (E) Relative |
|  | (C) Nurse | (F) Other…………… |
| 1. Date of Delivery |  | |
| 1. Date of discharge |  | |

Contact details of main home

| 1. House number |  |
| --- | --- |
| 1. Street name |  |
| 1. Village |  |
| 1. Region |  |
| 1. Phone number |  |
| 1. Name of next of kin at this residence |  |
| 1. Phone Number for next of kin |  |
| 1. Alternative phone number for reaching the woman besides next of kin number |  |
| 1. Good or appropriate time to call |  |

Contact details of residence after discharge from hospital

| 1. House number |  |
| --- | --- |
| 1. Street name |  |
| 1. Village |  |
| 1. Region |  |
| 1. Phone number |  |
| 1. Name of next of kin at this residence |  |
| 1. Phone number for next of kin |  |
| 1. Alternative phone number for reaching the woman besides next of kin number |  |
| 1. Description of how to find the woman’s residence |  |

| CASE IDENTIFICATION NUMBER |  |  |  |  |
| --- | --- | --- | --- | --- |

**Part 2**

- **This part should be completed only for women who have delivered in one of the study hospitals, or for women who have delivered at home but are admitted to the hospital for delivery of retained placenta.**
- **It should be completed once every 24 hours for all women who are post partum while in hospital**
- **A case identification number should be assigned for every woman when she is first seen after delivery in hospital. This number will be unique to her and would be used for follow up. The number assigned to her should be the same in part 1, 2 or 3 and the self reporting card.**
- **Part 1 and 2 of the case identification form should be retained and used to follow up women who have been discharged from the hospital throughout the entire period.**

**Section 2A:** Signs and symptoms questions

- **The asterisk (*) in the table below, denotes the days a phone call should be made if the woman is discharged from hospital.**
- **Phone calls will be made to follow up on the woman. In these instances;**
  1. **Please ask whether any of the symptoms listed in the table below occurred since the last phone call.**
  2. **If the woman is not available or busy, speak to the next of kin to determine when to call again. If there is no reply, try to call again twice, the same day and then the next day. If you still cannot speak to the woman, try calling the alternative number provided in Part 1; section C. After which, if you still cannot reach her through this number, try visiting.**
  3. **Speak to next of kin in the case of a referral or death**

| CASE IDENTIFICATION NUMBER |  |  |  |  |
| --- | --- | --- | --- | --- |

|  | **On admission** | **Date of delivery** | **1** | **2** | **3** | **4** | **5** | **6** | **7*** | **8** | **9** | **10** | **11** | **12** | **13** | **14*** | **15** | **16** | **17** | **18** | **19** | **20** | **21** |
| --- | --- | --- | --- | --- | --- | --- | --- | --- | --- | --- | --- | --- | --- | --- | --- | --- | --- | --- | --- | --- | --- | --- | --- |
| **Tick the number of days post partum** | **N/A** |  |  |  |  |  |  |  |  |  |  |  |  |  |  |  |  |  |  |  |  |  |  |
| **Does the woman complain of any of the following symptoms? Tick the symptoms as they appear on each postpartum day.** | | | | | | | | | | | | | | | | | | | | | | | |
| 1. **Hot/sweaty** |  |  |  |  |  |  |  |  |  |  |  |  |  |  |  |  |  |  |  |  |  |  |  |
| 1. **Nausea/vomiting** |  |  |  |  |  |  |  |  |  |  |  |  |  |  |  |  |  |  |  |  |  |  |  |
| 1. **Pain in pelvis, abdomen, perineal area or wound** |  |  |  |  |  |  |  |  |  |  |  |  |  |  |  |  |  |  |  |  |  |  |  |
| 1. **Discomfort during urination** |  |  |  |  |  |  |  |  |  |  |  |  |  |  |  |  |  |  |  |  |  |  |  |
| 1. **Offensive vaginal discharge** |  |  |  |  |  |  |  |  |  |  |  |  |  |  |  |  |  |  |  |  |  |  |  |
| 1. **Raised temperature** |  |  |  |  |  |  |  |  |  |  |  |  |  |  |  |  |  |  |  |  |  |  |  |
| 1. **Wound redness, swelling or opening** |  |  |  |  |  |  |  |  |  |  |  |  |  |  |  |  |  |  |  |  |  |  |  |
| 1. **None of the above** |  |  |  |  |  |  |  |  |  |  |  |  |  |  |  |  |  |  |  |  |  |  |  |
| 1. **Are you using any antibiotics? If yes, complete section B (antibiotics questions). If no, leave out section B** |  |  |  |  |  |  |  |  |  |  |  |  |  |  |  |  |  |  |  |  |  |  |  |
| 1. **Tick day of discharge** |  |  |  |  |  |  |  |  |  |  |  |  |  |  |  |  |  |  |  |  |  |  |  |
| 1. **Tick day of referral**   **State which hospital__________________** |  |  |  |  |  |  |  |  |  |  |  |  |  |  |  |  |  |  |  |  |  |  |  |
| 1. **Tick day of death** |  |  |  |  |  |  |  |  |  |  |  |  |  |  |  |  |  |  |  |  |  |  |  |

| CASE IDENTIFICATION NUMBER |  |  |  |  |
| --- | --- | --- | --- | --- |

|  | **On admission** | **Date of delivery** | **22** | **23** | **24** | **25** | **26** | **27** | **28** | **29** | **30** | **31** | **32** | **33** | **34** | **35** | **36** | **37** | **38** | **39** | **40** | **41** | **42*** |
| --- | --- | --- | --- | --- | --- | --- | --- | --- | --- | --- | --- | --- | --- | --- | --- | --- | --- | --- | --- | --- | --- | --- | --- |
| **Tick the number of days post partum** | **N/A** |  |  |  |  |  |  |  |  |  |  |  |  |  |  |  |  |  |  |  |  |  |  |
| **Does the woman complain of any of the following symptoms? Tick the symptoms as they appear on each postpartum day.** | | | | | | | | | | | | | | | | | | | | | | | |
| 1. **Hot/sweaty** |  |  |  |  |  |  |  |  |  |  |  |  |  |  |  |  |  |  |  |  |  |  |  |
| 1. **Nausea/vomiting** |  |  |  |  |  |  |  |  |  |  |  |  |  |  |  |  |  |  |  |  |  |  |  |
| 1. **Pain in pelvis, abdomen, perineal area or wound** |  |  |  |  |  |  |  |  |  |  |  |  |  |  |  |  |  |  |  |  |  |  |  |
| 1. **Discomfort during urination** |  |  |  |  |  |  |  |  |  |  |  |  |  |  |  |  |  |  |  |  |  |  |  |
| 1. **Offensive vaginal discharge** |  |  |  |  |  |  |  |  |  |  |  |  |  |  |  |  |  |  |  |  |  |  |  |
| 1. **Raised temperature** |  |  |  |  |  |  |  |  |  |  |  |  |  |  |  |  |  |  |  |  |  |  |  |
| 1. **Wound redness, swelling or opening** |  |  |  |  |  |  |  |  |  |  |  |  |  |  |  |  |  |  |  |  |  |  |  |
| 1. **None of the above** |  |  |  |  |  |  |  |  |  |  |  |  |  |  |  |  |  |  |  |  |  |  |  |
| 1. **Are you using any antibiotics? If yes, complete section B (antibiotics questions). If no, leave out section B** |  |  |  |  |  |  |  |  |  |  |  |  |  |  |  |  |  |  |  |  |  |  |  |
| 1. **Tick day of discharge** |  |  |  |  |  |  |  |  |  |  |  |  |  |  |  |  |  |  |  |  |  |  |  |
| 1. **Tick day of referral**   **State to which hospital_________________** |  |  |  |  |  |  |  |  |  |  |  |  |  |  |  |  |  |  |  |  |  |  |  |
| 1. **Tick day of death** |  |  |  |  |  |  |  |  |  |  |  |  |  |  |  |  |  |  |  |  |  |  |  |

| CASE IDENTIFICATION NUMBER |  |  |  |  |
| --- | --- | --- | --- | --- |

**Section 2B:** Antibiotic questions

- **This section should be completed only if question 9 in section 2A is YES**

| 1. **Were any antibiotics administered to the woman?** | |  | **(A)No** | **Terminate** |
| --- | --- | --- | --- | --- |
|  |  |  | **(B)Yes** |  |
| 1. **If yes, when was it given?** | |  | **(A)Ante-partum** | |
|  |  |  | **(B)Intra-partum** | |
|  |  |  | **(C)Post-partum** | |
| 1. **If antibiotics were in either state in (b), what was the route of administration?** | |  | **(A)Intravenous (IV)** | |
|  |  |  | **(B)Intramuscular (IM)** | |
|  |  |  | **(C)Oral. Skip Questions (4), (5) and go to (6)** | |
| 1. **If IV or IM routes were used, what antibiotics were administered?** | **(A) IV** |  | **(A1)Ceftriaxone** | |
|  |  |  | **(A2)Benzyl penicillin** | |
|  |  |  | **(A3)Gentamicin** | |
|  |  |  | **(A4)Others (specify)**  **……………………………………………………………** | |
|  | **(B) IM** |  | **(B1)Ceftriaxone** | |
|  |  |  | **(B2)Benzyl penicillin** | |
|  |  |  | **(B3)Gentamicin** | |
|  |  |  | **(B4) Other (specify)**  **…………………………………………………………..** | |
| 1. **How long were IM or IV antibiotics used?** | |  | **(A)1 day** | |
|  |  |  | **(B)2 days** | |
|  |  |  | **(C)3 days** | |
|  |  |  | **(D)Other (specify)**  **………………………………………………………….** | |
| 1. **Were oral antibiotics used?** | |  | **(A)Yes** | |
|  |  |  | **(B) No** | **Terminate** |
| 1. **If yes, were they continued shortly after IV and IM antibiotics?** | |  | **(A)Yes** | |
|  |  |  | **(B)No** | **Specify reason**  **………………………………………………..** |
| 1. **If yes, specify which oral antibiotic was given and the duration.** | |  | **Specify antibiotic given.**  **…………………………………………………………** | |
|  |  |  | **Specify duration**  **…………………………………………………………** | |
| 1. **What was the main indication for antibiotic use?** | |  | **(A)Sepsis** | |
|  |  |  | **(B)Prophylaxis** | |
|  |  |  | **(C)Possible signs of infection. State the sign. ………………………………………………………..** | |
|  |  |  | **(D)Other (specify) …………………………………………………………** | |

| CASE IDENTIFICATION NUMBER |  |  |  |  |
| --- | --- | --- | --- | --- |

**Part 3**

**In depth case identification form**

- **Ensure the case identification number for this woman is correct**

**(Note: If you are doing a home visit, obtain the number from the call centre)**

- **A new form of part 3 should be inserted in the woman’s folder, every time the woman answers ‘yes’ (whether in hospital or at home) to any of the screening questions in part 2, section A.**
- **This form will only be completed if the woman answers ‘yes’ to screening questions in part 2, section A.**

| 1. **Name of data collector** | **……………………………………………………** | |
| --- | --- | --- |
| 1. **Where is this form being completed?** |  | **(A)Hospital** |
|  |  | **(B)Woman’s home** |
|  |  | **(C)Other(please state):**  **……………………………………………** |
| 1. **Today’s date** | **…………………………………………………………**  **Day/month/year** | |
| 1. **Woman’s full name (first, husbands, family)** | **…………………………………………………………**  **…………………………………………………………** | |
| 1. **Name of hospital where delivery took place** | **…………………………………………………………** | |
| 1. **Date of delivery** | **…………………………………………………………**  **Day/month/year** | |
| 1. **Number of days post partum** | **…………………….......................days** | |
| 1. **Woman’s village/ town of residence** | **…………………………………………………………** | |
| 1. **Woman’s caste (use caste groups in DHS/Census)** | **…………………………………………………………** | |

| CASE IDENTIFICATION NUMBER |  |  |  |  |
| --- | --- | --- | --- | --- |

| 1. **Is this the first time that this woman has presented with these symptoms, or has she been seen for the same infection previously in this pregnancy?** |  | **(A)First time** |
| --- | --- | --- |
|  |  | **(B)Has been seen previously** |
|  |  | **(C)Don’t know** |
| 1. **What type of delivery occurred in this hospital? (May tick more than one)?** |  | **(A)Normal vaginal** |
|  |  | **(B)Vacuum** |
|  |  | **(C)Forceps** |
|  |  | **(D)Breech** |
|  |  | **(E)Twins** |
|  |  | **(F)Vaginal extraction of dead foetus** |
|  |  | **(G)Caesarean section** |
|  |  | **(H)Delivery of placenta only (e.g. retained placenta after home delivery)** |
| 1. **Is there a perineal/vaginal wound?** |  | **(A)No** |
|  |  | **(B)Yes: state**  **………………………………** |
|  |  | **(C)Episiotomy** |
|  |  | **(D)Tear** |
|  |  | **(E)Other, state ………………………………** |
| 1. **Who conducted the delivery of the baby?** |  | **(A)Doctor** |
|  |  | **(B)Midwife** |
|  |  | **(C)Nurse** |
|  |  | **(D)Dai** |
|  |  | **(E)Relative** |
|  |  | **(F)Other, please state**  **………………………………** |
| 1. **Who conducted the delivery of the placenta?** |  | **(A)Doctor** |
|  |  | **(B)Midwife** |
|  |  | **(C)Nurse** |
|  |  | **(D)Dai** |
|  |  | **(E)Relative** |
|  |  | **(F)Other, please state**  **………………………………** |
| 1. **Was this woman discharged home and then readmitted?** |  | **(A)Yes** |
|  |  | **(B)No** |
| 1. **If yes to above, please explain reason for readmission** | **..........................................**  **..........................................**  **..........................................** | |

| CASE IDENTIFICATION NUMBER |  |  |  |  |
| --- | --- | --- | --- | --- |

| **Does the woman currently have any of the following symptoms:** | | | | |
| --- | --- | --- | --- | --- |
| 1. **Pelvic pain** |  | | **(A)Yes** | |
|  |  | | **(B)No** | |
| 1. **Abdominal pain** |  | | **(A)Yes** | |
|  |  | | **(B)No** | |
| 1. **Nausea or vomiting** |  | | **(A)Yes** | |
|  |  | | **(B)No** | |
| 1. **Offensive, smelly vaginal discharge** |  | | **(A)Yes** | |
|  |  | | **(B)No** | |
| 1. **Any one of the following: Increased frequency, urgency, hesitancy dribbling, discharge or pain on urination** |  | | **(A)Yes** | |
|  |  | | **(B)No** | |
| 1. **Any one of the following: discharge, opening, bruising, redness, tenderness or swelling around a perineal wound** |  | | **(A)Yes** | |
|  |  | | **(B)No** | |
|  |  | | **No perineal wound** | |
| 1. **Any one of the following: : discharge, opening, bruising, redness, tenderness or swelling around the caesarean section wound** |  | | **(A)Yes** | |
|  |  | | **(B)No** | |
|  |  | | **No caesarean section wound** | |
| **Examine the woman, taking her temperature if a vital signs chart is not available. Can you detect any of the following signs?** | | | | |
| 1. **Temperature 38.5^o^C/101.3^o^F or higher on any occasion** | |  | | **(A)Yes** |
|  |  |  | | **(B)No** |
| 1. **Delay in the reduction of the size of the uterus (<2cm a day for the first 8 days)** | |  | | **(A)Yes** |
|  |  |  | | **(B)No** |
| 1. **Abdominal or pelvic tenderness** | |  | | **(A)Yes** |
|  |  |  | | **(B)No** |
| 1. **Abnormal (odorous or purulent) vaginal discharge** | |  | | **(A)Yes** |
|  |  |  | | **(B)No** |
| 1. **Any one of the following: discharge, opening, bruising, redness, tenderness or swelling around a perineal wound** | |  | | **(A)Yes** |
|  |  |  | | **(B)No** |
|  |  |  | | **No perineal wound** |
| 1. **Any one of the following: discharge, opening, bruising, redness, tenderness or swelling around the caesarean section wound** | |  | | **(A)Yes** |
|  |  |  | | **(B)No** |
|  |  |  | | **No caesarean section wound** |

| CASE IDENTIFICATION NUMBER |  |  |  |  |
| --- | --- | --- | --- | --- |

**Annex 6b – Women’s self reporting card**

**Women’s Self Reporting Card**

| CASE IDENTIFICATION NUMBER |  |  |  |  |
| --- | --- | --- | --- | --- |

|  | No of days post partum | Date of del. | 1 | 2 | 3 | 4 | 5 | 6 | 7 * | 8 | 9 | 10 | 11 | 12 | 13 | 14* | 15 | 16 | 17 | 18 | 19 | 20 | 21 |
| --- | --- | --- | --- | --- | --- | --- | --- | --- | --- | --- | --- | --- | --- | --- | --- | --- | --- | --- | --- | --- | --- | --- | --- |
| (Drawings) | Tick the number of days post partum |  |  |  |  |  |  |  |  |  |  |  |  |  |  |  |  |  |  |  |  |  |  |
|  | Hot/sweaty |  |  |  |  |  |  |  |  |  |  |  |  |  |  |  |  |  |  |  |  |  |  |
|  | Nausea/vomiting |  |  |  |  |  |  |  |  |  |  |  |  |  |  |  |  |  |  |  |  |  |  |
|  | Pain in pelvis, abdomen, perineal area or wound |  |  |  |  |  |  |  |  |  |  |  |  |  |  |  |  |  |  |  |  |  |  |
|  | Discomfort during urination |  |  |  |  |  |  |  |  |  |  |  |  |  |  |  |  |  |  |  |  |  |  |
|  | Offensive vaginal discharge |  |  |  |  |  |  |  |  |  |  |  |  |  |  |  |  |  |  |  |  |  |  |
|  | Raised temperature |  |  |  |  |  |  |  |  |  |  |  |  |  |  |  |  |  |  |  |  |  |  |
|  | Wound redness, swelling or opening |  |  |  |  |  |  |  |  |  |  |  |  |  |  |  |  |  |  |  |  |  |  |
|  | None of the above |  |  |  |  |  |  |  |  |  |  |  |  |  |  |  |  |  |  |  |  |  |  |
|  | Have you taken any antibiotics today? |  |  |  |  |  |  |  |  |  |  |  |  |  |  |  |  |  |  |  |  |  |  |

| CASE IDENTIFICATION NUMBER |  |  |  |  |
| --- | --- | --- | --- | --- |

**Note: This information should appear on the back side of the self reporting card.**

For each day after your baby is born tick the form on the first row to show how many days it has been since your baby was born. Then check to see if you have any of the following symptoms. Are you:

- **hot and sweat**
- **sick or are vomiting,**
- **Having any pain in your pelvis, abdomen, the perineal area or around the caesarean section wound**
- **Do you feel uncomfortable when passing urine**
- **Having an offensive and smelly vaginal discharge**
- **Having a raised temperature**
- **Having wound redness, swelling or opening (around a caesarean section or episiotomy wound)**

If you have any of these symptoms then tick the box which corresponds to the symptom and the day. If you don’t have any symptoms on that day just tick the box in the ‘none of the above’ row.

If you begin to experience any of the symptoms listed it is very important you call the data collector at the hospital you delivered, his/her number is given below

The data collector you met in the hospital will also try and contact you on the following days after your baby is born – day 7, 14 and 42.

Free phone telephone number here (Data Collector’s number)

| CASE IDENTIFICATION NUMBER |  |  |  |  |
| --- | --- | --- | --- | --- |

|  | No of days post partum | Date of del. | 22 | 23 | 24 | 25 | 26 | 27 | 28 | 29 | 30 | 31 | 32 | 33 | 34 | 35 | 36 | 37 | 38 | 39 | 40 | 41 | 42 * |
| --- | --- | --- | --- | --- | --- | --- | --- | --- | --- | --- | --- | --- | --- | --- | --- | --- | --- | --- | --- | --- | --- | --- | --- |
| (Drawings) | Tick the number of days post partum |  |  |  |  |  |  |  |  |  |  |  |  |  |  |  |  |  |  |  |  |  |  |
|  | Hot/sweaty |  |  |  |  |  |  |  |  |  |  |  |  |  |  |  |  |  |  |  |  |  |  |
|  | Nausea/vomiting |  |  |  |  |  |  |  |  |  |  |  |  |  |  |  |  |  |  |  |  |  |  |
|  | Pain in pelvis, abdomen, perineal area or wound |  |  |  |  |  |  |  |  |  |  |  |  |  |  |  |  |  |  |  |  |  |  |
|  | Discomfort during urination |  |  |  |  |  |  |  |  |  |  |  |  |  |  |  |  |  |  |  |  |  |  |
|  | Offensive vaginal discharge |  |  |  |  |  |  |  |  |  |  |  |  |  |  |  |  |  |  |  |  |  |  |
|  | Raised temperature |  |  |  |  |  |  |  |  |  |  |  |  |  |  |  |  |  |  |  |  |  |  |
|  | Wound redness, swelling or opening |  |  |  |  |  |  |  |  |  |  |  |  |  |  |  |  |  |  |  |  |  |  |
|  | None of the above |  |  |  |  |  |  |  |  |  |  |  |  |  |  |  |  |  |  |  |  |  |  |
|  | Have you taken any antibiotics today? |  |  |  |  |  |  |  |  |  |  |  |  |  |  |  |  |  |  |  |  |  |  |

| CASE IDENTIFICATION NUMBER |  |  |  |  |
| --- | --- | --- | --- | --- |

**Note: This information should appear on the back side of the self reporting card.**

For each day after your baby is born tick the form on the first row to show how many days it has been since your baby was born. Then check to see if you have any of the following symptoms. Are you:

- **hot and sweat**
- **sick or are vomiting,**
- **Having any pain in your pelvis, abdomen, the perineal area or around the caesarean section wound**
- **Do you feel uncomfortable when passing urine**
- **Having an offensive and smelly vaginal discharge**
- **Having a raised temperature**
- **Having wound redness, swelling or opening (around a caesarean section or episiotomy wound)**

If you have any of these symptoms then tick the box which corresponds to the symptom and the day. If you don’t have any symptoms on that day just tick the box in the ‘none of the above’ row.

If you begin to experience any of the symptoms listed it is very important you call the data collector at the hospital you delivered, his/her number is given below

The data collector you met in the hospital will also try and contact you on the following days after your baby is born – day 7, 14 and 42.

Free phone telephone number here (Data Collector’s number)

**Annex 6c – Supervision checklist**

**Supervision Checklist**

**Supervision checklist [for use during fortnight/ monthly visits by supervisor or RF]**

| 1. Date of supervisory visit | Day/month/year | |
| --- | --- | --- |
| 1. Hospital/district name |  | |
| 1. Find out numbers of births in hospital for the previous month | State month data pertains to | |
| 1. Look up delivery and theatre register. Count numbers a, b, c, d, e, f (adjacent column) for the month and record these here | 1. Live births |  |
|  | 1. Still births |  |
|  | 1. Total deliveries |  |
|  | 1. Vaginal deliveries (unassisted) |  |
|  | 1. Instrumental (forceps, vacuum) |  |
|  | 1. Caesarean section |  |

| 1. Collect the case identification forms completed for the previous month. Record the numbers of cases identified here | Numbers of case identification forms with parts 1 and 2 completed for this month |  |
| --- | --- | --- |
|  | Number of NPFs completed for this month |  |
|  | Does the number of case identification forms with parts 1 and two completed plus the number of NPF forms completed for this month equal the number of deliveries (see question 4 part c)? If not, find out the reason and encourage data collectors to improve data capture | Yes |
|  |  | No |
|  | Numbers of fully completed forms (women with infection) in hospital |  |
|  | Numbers of fully completed forms (women with infection) from community |  |
| 1. Go to the ward and identify a woman with a puerperal infection, if possible. Find the relevant case identification form and check if it is accurately completed. If there are no women with infections, pick a few forms and check its completion with the data collector, pointing out problems if there are any | | |
| 1. Conduct key informant interviews using provided topic guide | | |
| 1. Record any notes or problems for discussion with the other members of the research team below and overleaf | | |

**Annex 7: Qualitative KII consent form**

**Consent Form**


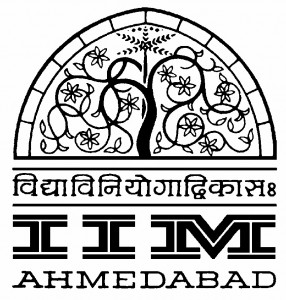


**
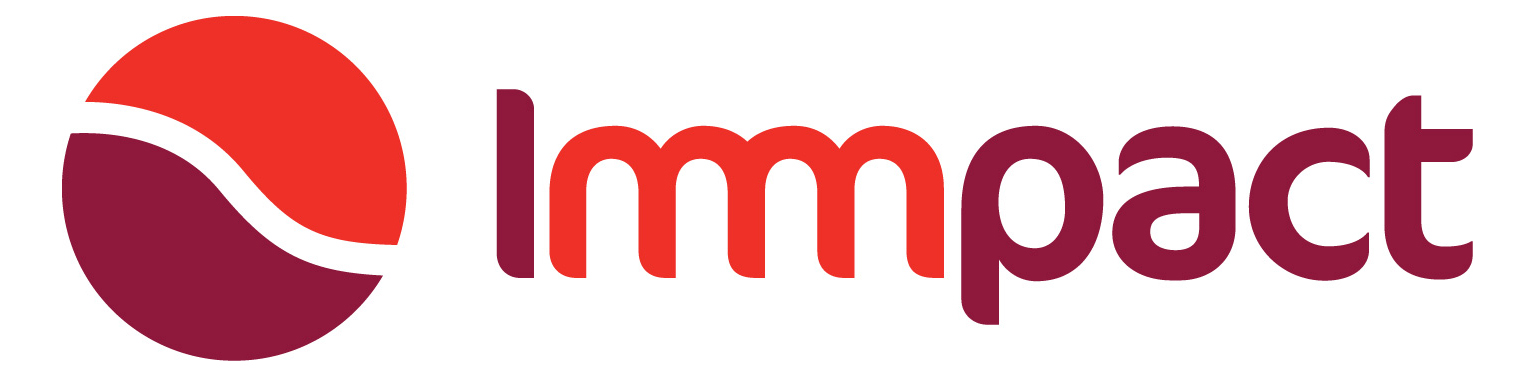
**

**An infection control intervention study: Using infection control as an entry point for improving the quality of delivery care and strengthening health systems in developing countries.**

Name of Key Informant­­­­­­­­­­­­­­­­­­­­­­______________________________________________

The purpose of this study has been explained to me and I am happy to take part. I am aware that any information about me will be held in confidence and in accordance with the sixth revision (2008) of the Declaration of Helsinki.

Signed (Key Informant)­­­­­­­­­­­____________________________ Date______________

Name of Interviewer ________________________________________________

Signed (Interviewer)­­­­­­­­­­­­­­_______________________________Date______________

**Annex 8 – Qualitative instrument – Key informant topic guide**

**Qualitative Instrument**

**Key Informant Interview Topic Guide**

**Topic guide for key informant interviews**

**BACKGROUND (please refer to annex 3 for more background information)**

A “health system consists of all organisations, people and actions whose primary intent is to promote, restore or maintain health”

According to the WHO a health system is made up of six parts:

1. **Service Delivery:**

Delivery of effective, safe, good quality personal and non personal health interventions to those that need them, when and where needed, with the minimum waste of resources.

1. **Health Workforce (HR):**

“All people engaged in actions whose primary intent is to enhance health”. This definition includes clinical staff, such as nurses and midwives as well management and support staff (such as accountants and porters).

1. **Information:**

Production, analysis, dissemination and use of timely and reliable information on health determinants, health systems performance and health status

1. **Medical Products, Vaccines and Technologies:**

Equitable access to essential medical products, vaccines and technologies of assured quality, safety, efficacy and cost-effectiveness and their scientifically sound and cost-effective use

1. **Financing:**

Function of the health system concerned with mobilisation, accumulation and allocation of money to cover the health needs of the people

1. **Leadership and Governance**

The way stakeholders in the health system finance, deliver, monitor and use health services are managed, so that strategic policy frameworks exist and are combined with effective oversight, coalition building, the provision of appropriate regulations and incentives, attention to system-design and accountability.

The Key Informant in-depth interviews will be used to assess the relative strength of the health system. This will be done by assessing the 6 individual components of a health system, according to the WHO definition.^2^

KI interviews are open-ended. What is given here is a topic guide for different types of stakeholders. Please explore around these issues and ask intelligent follow-up questions when interesting points are made. If the interviewee is making interesting points which are relevant to the evaluation question, please continue, even if they don’t fall under one of these topics!

(You often find out the questions you *should have* asked in the course of the interview – these can be incorporated in later interviews. Similarly, important points raised by one person can be put to another for comment/agreement/disagreement, when relevant.)

Make sure you discuss all of the topics, but some will be more fruitful than others, so don’t worry if they have little to say on some of the issues.

Each question is numbered, after which some questions have letters, these act as an indication of the type of information each question is trying to elicit. Depending on the question it may or may not be appropriate to ask these sub-questions - the interviewer should be wary of leading the informant. Please ask your respondents the questions in ALL the sections. Although not all your respondents may be comfortable answering all your questions, leave this to their discretion and explain in your notes why some sections are not completed if this situation occurs.

It is useful to write summary points as you are interviewing, but for a full verbatim account, with accurate quotes, a back-up recording is needed.

**TOPIC GUIDE**

The topic guide has been revised and is found as a separate document i.e. the qualitative study protocol (V. 29^th^ July 10) – **using infection control as an entry point for improving the quality of delivery care and strengthening health systems in developing countries.**

1. During the preliminary visit to IIM in April 2010 it became clear that data on infections were not routinely recorded in government hospitals, which makes comparisons between different hospitals difficult during the site selection process. Also with institutional delivery rates increasing so rapidly in Gujarat, the number of deliveries in a given hospital in one year may not be an accurate predictor of the number of deliveries the next year. [↑](#footnote-ref-2)
2. We plan to have government hospitals as our study sites unless we find Non-governmental (NGO) hospitals of sufficient size. [↑](#footnote-ref-3)
3. Britten, N. (1995). Qualitative Interviews in Medical Research. *British Medical Journal,* **311** 251-253

   DiCicco-Bloom, B. & Crabtree, B. F. (2006) The Qualitative Research Interview *Medical Education*, **40** 314–321 [↑](#footnote-ref-4)
4. Must answer yes to 1a only or Yes to 1b and 1c [↑](#footnote-ref-5)
5. Must at least have one of **2a-c** and answer yes to **3 & 4** [↑](#footnote-ref-6)
6. Must answer yes to **5 ,6** and **7** **Or** Yes to **5**, and yes to either **6 or 7** and yes to two or more of **7a-e** [↑](#footnote-ref-7)
7. Must answer yes to **8**, and yes to at least two of **9a-c** **Or** Yes to **8,** yes to one of **9a-c** and yes to at least 2 of **10a-d** [↑](#footnote-ref-8)
8. Must answer yes to 11 and yes to both 12a and 12b

   Or

   Yes to 11, yes to one of 12a or 12b, and yes to two of 13a-d

   Or

   Yes to 11, yes to none of 12a-b and yes to three or more of 13a-d* [↑](#footnote-ref-9)
